# Micro/Nanoscale Acoustic Manipulation: From Particle Control to Autonomous Microswimmers

**DOI:** 10.1002/advs.77557

**Published:** 2026-09-03

**Authors:** Jiahui Chu, Lemin Zhang, Xu Wang, Wenzong Li, Yahua Liu

**Affiliations:** ^1^ State Key Laboratory of High‐performance Precision Manufacturing Dalian University of Technology Dalian P. R. China; ^2^ Department of Anaesthesiology Central Hospital of Dalian University of Technology Dalian P. R. China

**Keywords:** acoustic manipulation, acoustic radiation forces, acoustic streaming, acoustically driven microswimmer, particle control

## Abstract

Acoustic manipulation and propulsion technologies have emerged as powerful platforms for micro/nanoscale control, enabled by their non‐contact operation, label‐free compatibility, biocompatibility, and strong penetration capability. This review systematically examines the physical mechanisms and recent advances in acoustically enabled passive particle manipulation and autonomous microswimmer propulsion. First, this article outlines the generation and propagation of acoustic waves and their fundamental interactions with matter, with emphasis on acoustic radiation forces and acoustic streaming. It then classifies representative device architectures and acoustic field modulation strategies based on bulk acoustic wave and surface acoustic wave systems. Building on this framework, recent progress in precise target manipulation across diverse media is synthesized, encompassing trapping, transport, enrichment, separation, and patterning. Furthermore, the review highlights the rapidly evolving field of acoustically driven microswimmers, analyzing autonomous propulsion mechanisms arising from shape‐ and density‐induced asymmetries, cavitation phenomena, and sharp‐edge oscillations, as well as recent advances in collective behavior, swarm coordination, and intelligent navigation. By delineating the developmental trajectory and key challenges of acoustic manipulation and propulsion, this review establishes a comprehensive knowledge framework for interdisciplinary researchers and highlights future opportunities in precision medicine, smart materials, micro/nano‐fabrication, and cross‐scale biological manipulation.

## Graphical abstract

This conceptual framework illustrates acoustic technology as a multifunctional platform enabling passive particle manipulation across scales and autonomous propulsion of microswimmers.

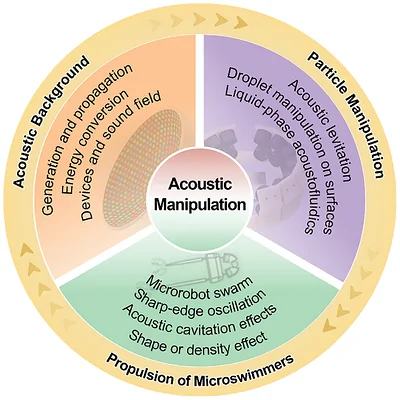

## 1 Introduction

Non‐invasive and high‐precision manipulation of microscopic objects, including microdroplets, biological cells and nanoparticles, is a foundational capability in modern science and engineering underpinning advances in material assembly [1, 2, 3], targeted therapy [4, 5], and micro/nanorobotics [6, 7]. Among available physical fields [8, 9], such as optical, magnetic, and electric fields, acoustic fields offer a distinctive combination of advantages [10, 11]. Their biocompatibility and deep penetration enable potential in vivo applications [12, 13], while manipulation mechanisms based on intrinsic material properties allow label‐free identification and handling [14]. Moreover, the continuously tunable wavelength over a broad frequency range supports versatile manipulation across multiple length scales, from millimeters to submicron dimensions [15]. Together, these features position acoustic technologies as a powerful and multifunctional platform for micro/nanoscale control.

The scope of acoustic manipulation has expanded dramatically, from early demonstrations of macroscopic levitation in air [16, 17, 18] to contemporary applications in non‐destructive cell sorting [19, 20], precise assembly in complex liquids [21, 22], and the realization of microrobots capable of intelligent navigation [23]. This versatility arises from two fundamental nonlinear mechanical effects governing acoustic–matter interactions: acoustic radiation forces and acoustic streaming [24, 25, 26]. Acoustic radiation forces, originating from momentum transfer, enable particle trapping, translation, and pattern formation, whereas acoustic streaming, driven by viscous energy dissipation, generates steady flows that can be exploited for mixing, pumping, and propulsion. Together, these mechanisms form the physical foundation of acoustic manipulation and propulsion.

Although numerous reviews have surveyed the broad applications of acoustic manipulation and propulsion [27, 28, 29, 30, 31, 32, 33], most are organized around specific device types or application scenarios (Table 1), often obscuring the underlying physical continuity from fundamental principles to diverse systems and functions. This review seeks to connect acoustic field generation, force–flow conversion, passive manipulation functions, and active microswimmer design rules within a unified, mechanism‐centered framework (Figure 1) [11, 34, 35, 36, 37]. We begin by presenting the core theory of acoustic wave generation, propagation, and energy conversion. We then analyze representative acoustic device architectures, categorizing their operating principles and field modulation strategies. Within this framework, we synthesize strategies and applications for passive, precise target manipulation across diverse media, including trapping, transport, enrichment, separation, and patterning. Finally, we focus on the rapidly evolving field of acoustically driven microswimmers, elucidating the physical mechanisms of autonomous propulsion, recent advances in collective and intelligent behaviors, and their emerging potential in biomedicine. By delineating the developmental trajectory and identifying key challenges, this review aims to provide a coherent foundation for interdisciplinary researchers and to stimulate future innovation across scientific and technological domains.

**TABLE 1:** Scope of representative prior reviews and the unique contribution of this work.

Prior review	Core scope & organizing principle	Unique contribution of our review
Yang et al., 2025 *Nat. Rev. Methods Primers* [29]	Classified by device topology and operational function, with an emphasis on biomedical applications	Reorganizes operational functions under a mechanism‐centered framework, unifying passive manipulation and active propulsion through a shared physical language
Ju et al., 2025 *ACS Nano* [6]	Broad interdisciplinary roadmap covering diverse actuation modalities	Delivers acoustic‐specific depth within a unified, mechanism‐centered framework
Guerassimoff et al., 2025 *Adv. Drug Deliv. Rev*. [32]	Focuses on single‐beam tweezers design and carrier engineering for targeted therapeutics	Distinguishes passive acoustophoretic carriers from active microswimmers, establishing physical criteria for selecting between strategies in drug delivery
Wu et al., 2024 *Adv. Drug Deliv. Rev*. [23]	Organized by swimmer design (mechanisms, fabrication, materials, and biomedical applications)	Provides the underlying physical foundation while systematically contrasting the design principles governing distinct swimmer classes
Liu et al., 2023 *Ultrason. Sonochem*. [33]	Sub‐MHz acoustic wave micromanipulation, focusing on low‐frequency, low‐cost acoustic sources	Integrates sub‐MHz platforms alongside MHz–GHz systems within a frequency–size–mechanism framework, clarifying how frequency dictates dominant physical mechanisms and operational regimes
Rufo et al., 2022 *Nat. Rev. Methods Primers* [28]	Device‐centric classification of biomedical acoustofluidics, emphasizing point‐of‐care diagnostics	Connects biomedical acoustofluidics to fundamental force/flow duality and extends this framework to autonomous microswimmer propulsion
Li et al., 2022 *Adv. Funct. Mater*. [30]	Focuses on propulsion mechanisms and fabrication techniques for microswimmers	Bridges the gap between passive acoustic manipulation and active propulsion, detailing fundamental design trade‐offs across swimmer architectures

**FIGURE 1:**
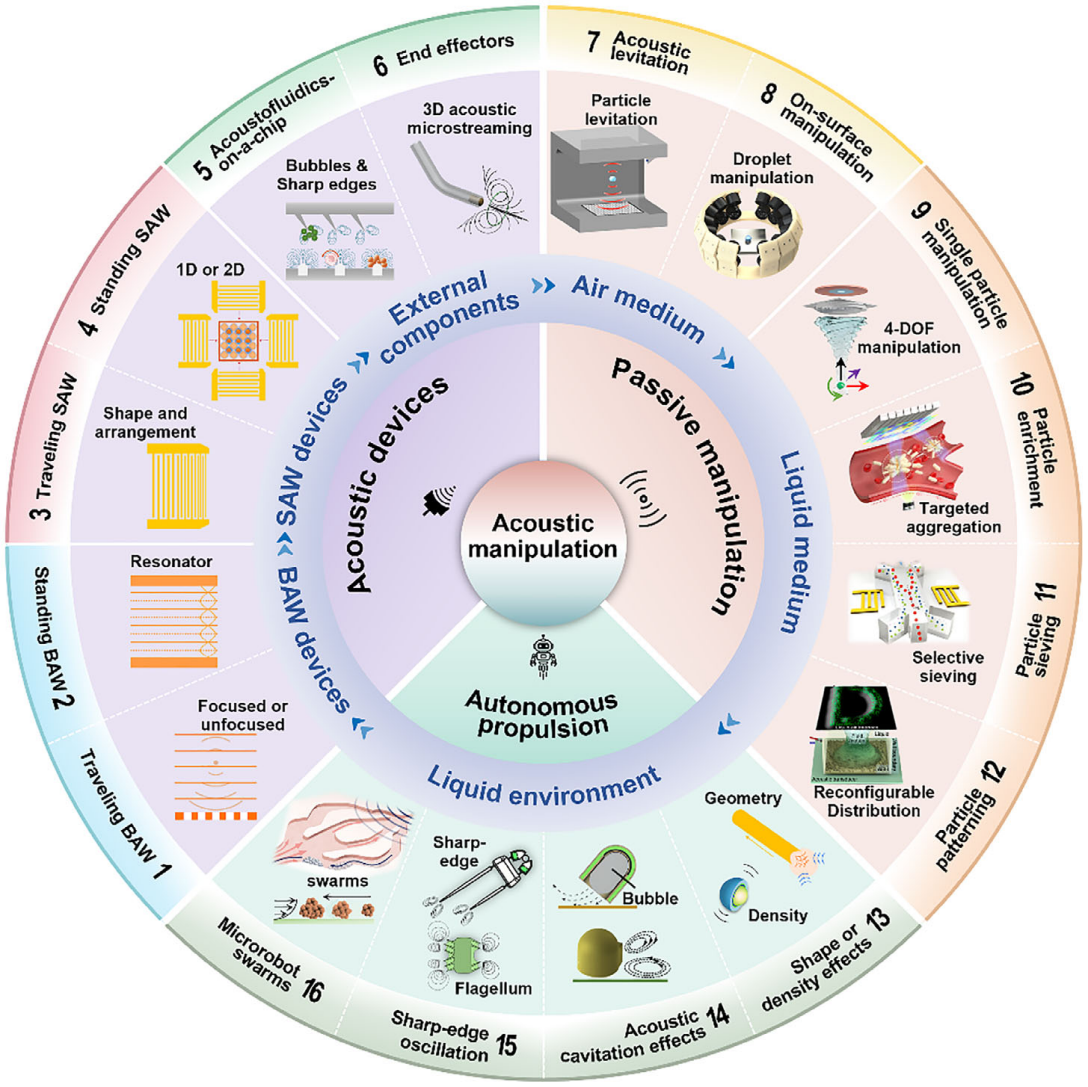
Conceptual framework of acoustic manipulation and propulsion. Acoustic technology, with its unique integrated advantages, has evolved into a powerful multifunctional platform widely applicable for passive manipulation of particles across media and scales, as well as driving microswimmers. On‐surface manipulation: Reproduced with permission [34]. Copyright 2023, AAAS; Single particle manipulation: Reproduced with permission [35]. Copyright 2024, AAAS; Particle enrichment: Reproduced with permission [36]. Copyright 2023, Springer Nature; Particle sieving: Reproduced with permission [11]. Copyright 2020, Annual Reviews; Particle patterning: Reproduced with permission [37]. Copyright 2020, American Chemical Society.

## 2 Acoustic Background: Theoretical Basis and Working Principles

### 2.1 Acoustic Wave Generation and Propagation

Acoustic waves arise from the coupled propagation of mechanical vibrations in elastic media. In most acoustic systems, they are generated via the inverse piezoelectric effect: piezoelectric transducers convert electrical signals into mechanical vibrations that radiate acoustic waves into adjacent solid or fluid media. Depending on transducer configuration and propagation characteristics, acoustic waves are broadly categorized into bulk acoustic waves (BAWs) and surface acoustic waves (SAWs).

BAWs are commonly excited using thickness vibration modes of piezoelectric ceramics or single crystals (e.g., PZT, LiNbO_3_, quartz), producing longitudinal waves that propagate through the entire working chamber (Figure 2A). At the microscopic level, BAWs originate from periodic oscillations of particles about their equilibrium positions; macroscopically, they manifest as spatiotemporal variations in acoustic pressure *p*, density ρ, particle velocity *u*, and displacement ξ [14]. In contrast, SAWs propagate along the surface of piezoelectric substrates—most commonly LiNbO_3_—and are excited using interdigital transducers (IDTs) with interleaved electrode fingers (Figure 2B) [38, 39]. The resonance frequency *f* of a standard IDT is determined by the IDT period λ (the distance between two adjacent fingers of the same polarity) as [40]

(1) $$f=\frac{\upsilon }{\lambda }\;$$

where υ is the SAW velocity in the piezoelectric substrate. By tailoring IDT geometries, the phase and amplitude of acoustic wavefronts can be precisely modulated, enabling the generation of complex fields [41].

**FIGURE 2:**
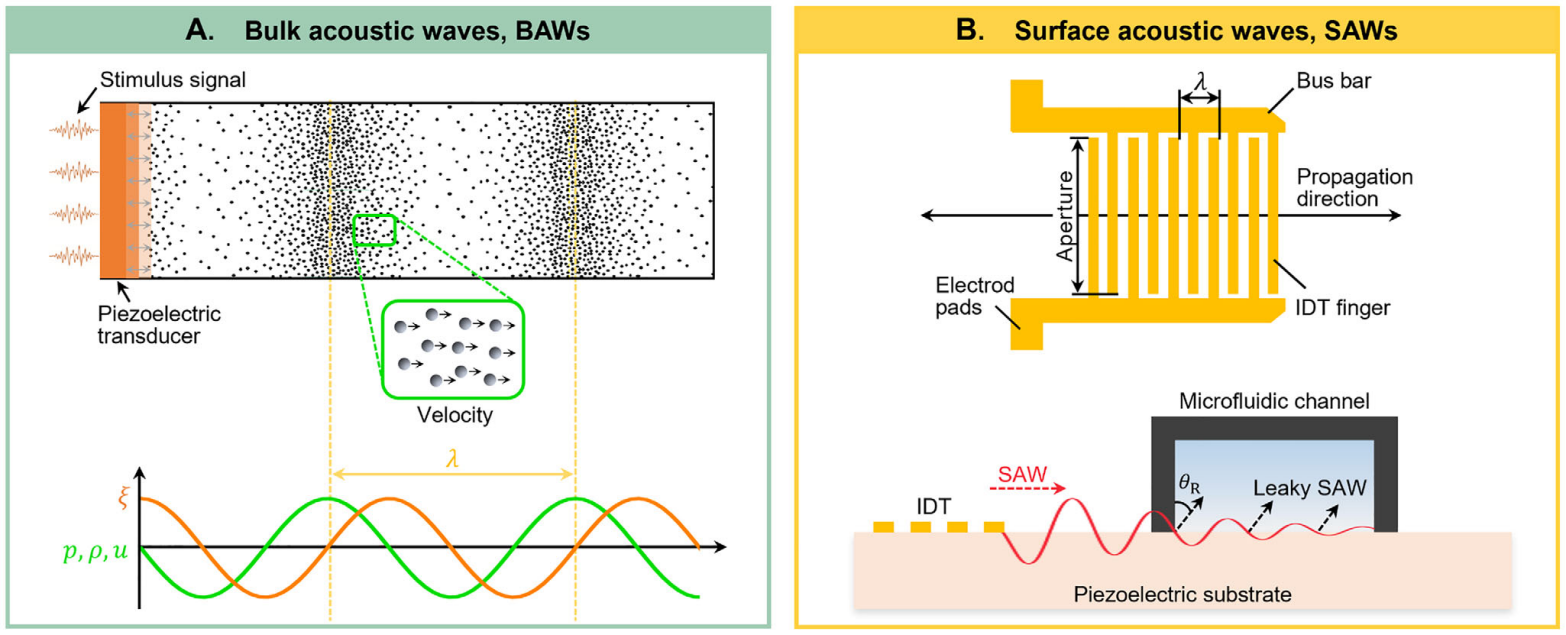
Generation and propagation characteristics of two types of acoustic waves. (A) Bulk acoustic waves are mechanical waves of compression and rarefaction in a medium. Within the wave, the local pressure *p*, density ρ, particle velocity *u*, and particle displacement ξ vary as a function of time and position, with specific relationships between these variables. Reproduced with permission [14]. Copyright 2022, American Chemical Society. (B) A surface acoustic wave device consisting of comb‐like electrodes, bus bars, and electrode pads on a piezoelectric substrate. The generated traveling waves propagate along the substrate surface and radiate pressure waves into the fluid medium at the Rayleigh angle θ_R_.

Acoustic frequencies span a wide spectrum—from audible sound (20 Hz–20 kHz) to ultrasound (>20 kHz), and even gigahertz regimes [42]. High‐frequency ultrasound characterized by short wavelengths and concentrated energy, is particularly effective for microscale manipulation. During propagation, acoustic energy is attenuated by viscous dissipation and molecular relaxation [43], resulting in an exponential decay of pressure amplitude *p* with distance *l*

(2) $$p=p_{0}\;e^{-\alpha l}$$

where *p*_0_ is the initial pressure amplitude and α the attenuation coefficient. The attenuation coefficient α is frequency‐dependent, typically scaling as α ∝ ω^2^ (here, ω is the angular frequency of the acoustic wave) in the viscous regime and exhibiting more complex behavior in relaxing media. Acoustic attenuation thus increases strongly with frequency, imposing practical limits on penetration depth at high frequencies.

### 2.2 Acoustic Energy Conversion

The functional utility of acoustic systems in manipulation and propulsion arises primarily from two nonlinear effects: acoustic radiation forces and acoustic streaming. Both originate from spatial gradients in acoustic energy density caused by wave scattering, reflection, and attenuation.

#### 2.2.1 Acoustic Radiation Forces

When an acoustic field interacts with suspended particles, momentum transfer from scattered waves generates acoustic radiation forces [44, 45, 46, 47, 48, 49, 50]. These forces are typically classified as primary radiation force, acting on individual particles, and secondary radiation force, arising from interactions between particles or between particles and boundaries. For a spherical particle with radius *a* in the Rayleigh limit (*a*  ≪  λ), suspended in a dilute, inviscid fluid and subjected to a time‐averaged harmonic acoustic field far from boundaries, the time‐averaged primary acoustic radiation force can be expressed as the gradient of Gor'kov's potential *U*_rad_ [51]

(3) $$F_{\mathrm{rad}}=-\nabla U_{\mathrm{rad}}$$

where

(4) $$U_{\mathrm{rad}}=\frac{4\pi a^{3}}{3}\;\left[\frac{1}{2}\kappa_{0}\langle p_{\mathrm{in}}^{2}\rangle f_{1}-\frac{3}{4}\rho_{0}\langle \nu_{\mathrm{in}}^{2}\rangle f_{2}\right]$$

Here, 4π*a* ^3^/3 is the volume of the particle, and the scattering coefficients are given by

(5) $$f_{1}=1-\tilde{\kappa }\text{, with}\;\tilde{\kappa }=\frac{\kappa_{p}}{\kappa_{0}}\;$$

(6) $$f_{2}=\frac{2\left(\tilde{\rho }-1\right)}{2\tilde{\rho }+1}\;\text{, with}\;\tilde{\rho }=\frac{\rho_{p}}{\rho_{0}}\;$$

Here, *f*_1_ and *f*_2_ are dimensionless scattering coefficients, ρ_0_ is the medium density, *p*_in_ and ν_in_ are the pressure and velocity in the wave at the point where the particle is located, κ_p_ and $\kappa_{0}=1/\left(\rho_{0}c_{0}^{2}\right)$ are the compressibility of the particle and the medium, respectively, ρ_p_ is the density of the particle, and *c*_0_ is the speed of sound in the liquid.

When two plane acoustic waves of equal wavelength and amplitude propagate in opposite directions, their interference generates stationary planes comprising pressure antinodes (points of maximum pressure) and pressure nodes (points of zero pressure). The primary radiation force can be subdivided into axial and transverse components within the standing wave fields [52]. The axial force acts in the direction of the propagation of the acoustic wave field and is stronger than the transverse force (Figure 3A) [53]. The axial acoustic radiation force acting on a compressible spherical particle in an inviscid fluid can be defined by the following equation [54]

(7) $$F_{\mathrm{rad}}=-\left(\frac{\pi p_{\mathrm{in}}^{2}V_{p}\kappa_{0}}{2\lambda }\right)\phi \sin\left(\frac{4\pi x}{\lambda }\right)$$

(8) $$\phi =\frac{5\rho_{p}-2\rho_{0}}{2\rho_{p}+\rho_{0}}\;-\frac{\kappa_{p}}{\kappa_{0}}$$

**FIGURE 3:**
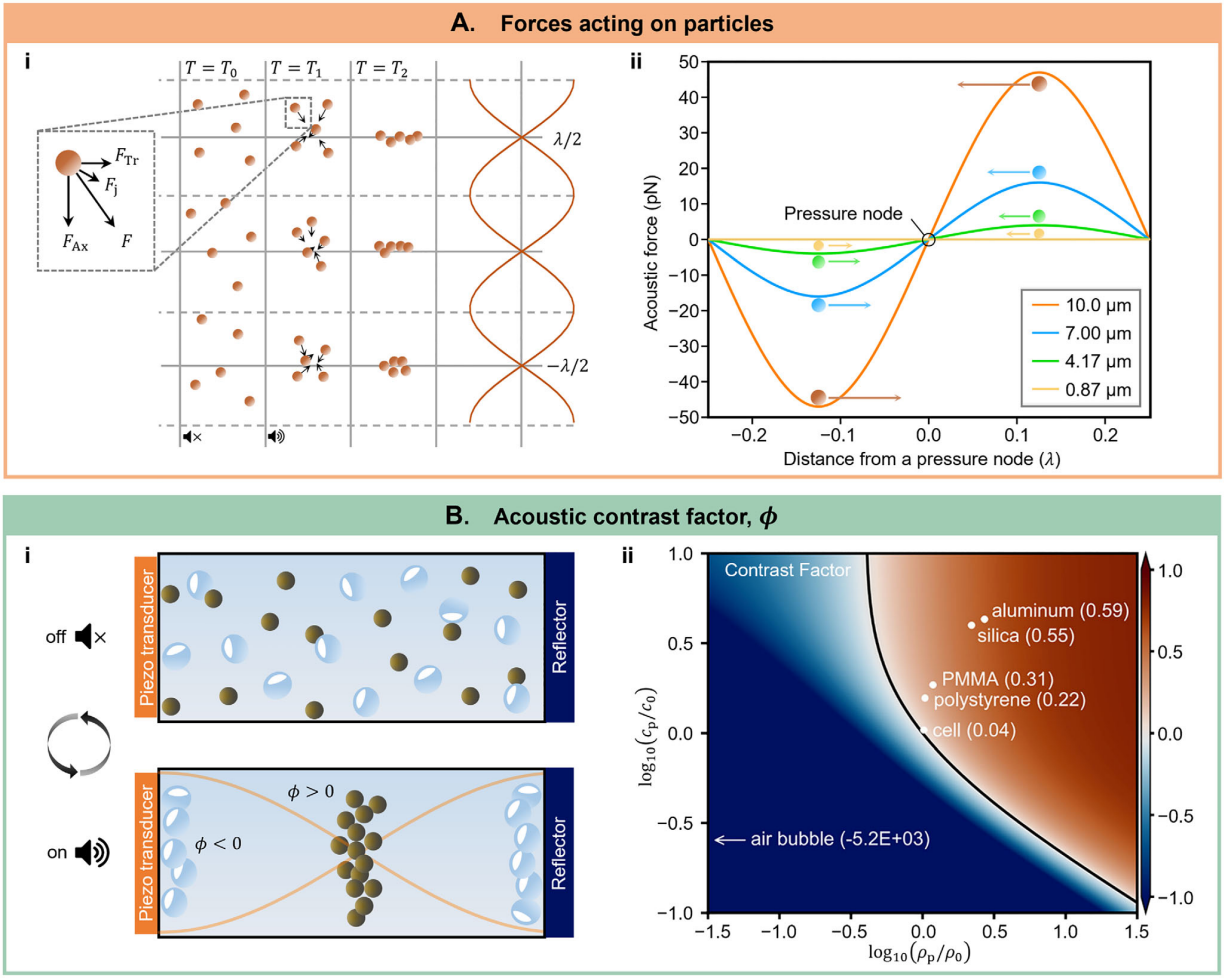
(A) Forces acting on particles in a standing wave field. (i) Acoustic forces that act on a particle: *F*_Ax_ is the axial component of the primary radiation force, *F*_Tr_ is the transverse component, and *F*_j_ are the interparticle forces (Bjerknes forces). At time *T*_1_ the acoustic forces have just begun to act on the particles and by time *T*_2_ a steady state has been reached. Reproduced with permission [52]. Copyright 2007, Royal Society of Chemistry. (ii) Acoustic radiation force distribution acting on polystyrene particles of different diameters at different positions in a standing wave field. Reproduced with permission [53]. Copyright 2009, Royal Society of Chemistry. (B) Differences in behavior between particles with varying acoustic contrast factors ϕ. (i) Illustrated cross‐section of a microfluidic channel showing negative ϕ particles collected in the pressure antinodes and positive ϕ particles in the pressure nodes. (ii) Acoustic contrast factor as a function of particle and fluid properties, with specific values plotted for common acoustic materials in water. Reproduced with permission [14]. Copyright 2022, American Chemical Society.

Here, *V*_p_, λ, and *x* are the volume of the particle, acoustic wave length, and distance from a pressure node, respectively. The acoustic contrast factor ϕ determines whether particles migrate toward pressure nodes or pressure antinodes according to their density ρ_p_ and compressibility κ_p_ relative to the surrounding medium. Specifically, particles with ϕ > 0 migrate toward pressure nodes, whereas those with ϕ < 0 migrate toward pressure antinodes (Figure 3B(i)) [52].

In addition to the primary acoustic radiation force resulting from momentum transfer from the primary acoustic field to the particles, an additional attractive or repulsive force—termed the secondary acoustic radiation force—arises from the interaction between waves scattered by adjacent particles [55]. This secondary force becomes significant only when particles are in close proximity or exhibit high compressibility. Specifically, for compressible microbubbles in aqueous solutions, the secondary acoustic radiation force is also known as the secondary Bjerknes force, first described by Vilhelm Bjerknes in 1906 [56]. Due to their high compressibility, microbubbles can undergo strong oscillations in response to the acoustic field (Figure 3B(ii)). These oscillations lead to an amplification of the scattered secondary waves. For the case of two oscillating bubbles, the secondary Bjerknes force *F*_j_ can be given by [57]

(9) $$F_{j}=-\;\langle V_{1}\nabla P_{2}\rangle =\frac{\rho_{0}}{4\pi d^{2}}\;\langle \frac{\partial V_{1}}{\partial t}\cdot \frac{\partial V_{2}}{\partial t}\rangle$$

where *V*_1_ and *V*_2_ are the volumes of the two bubbles, ∇*P*_2_ is the pressure gradient generated by the second bubble, *d* is the distance between the bubbles, and the angular brackets 〈〉 represent the time average during one oscillation time period. For bubble clusters, the secondary Bjerknes force is strongly size‐dependent, peaking near the Minnaert resonance frequency [57], where volumetric oscillations are maximally amplified. Its direction (attractive or repulsive) is determined by the relative oscillation phase between neighboring bubbles and can be tuned by adjusting the driving frequency, bubble size ratio, and inter‐bubble distance. This size‐dependent tunability provides the physical foundation for the programmable assembly and navigation of bubble‐based microswimmer swarms (Section 4.4).

For rigid or weakly compressible particles (e.g., polymer beads, cells), the secondary radiation force follows a distinct scaling relationship. When the interparticle distance *d* is much larger than the particle radius *a* but remains smaller than the acoustic wavelength (*a*  ≪  *d*  ≪  λ), the axial and transverse components scale as *a* ^6^/*d* ^4^ and *a* ^6^/*d* ^2^, respectively [26, 58]. This strong *a* ^6^ dependence leads to highly size‐selective interparticle interactions: particles larger than a few micrometers can undergo aggregation into ordered clusters or chains, whereas submicron particles experience negligible secondary forces and remain dispersed unless confined by strong acoustic potential wells.

Particle size also fundamentally limits manipulation resolution (Section 3.3.4). Although the spatial periodicity of the trapping potential in standing‐wave tweezers is determined by the acoustic wavelength (λ/2), single‐particle trapping additionally depends on the particle diameter *d*_p_ relative to the wavelength. Effective particle isolation requires λ/*d*_p_  ≈  3.2–3.6 to avoid multiple particles being captured within a single potential well [59]. For subwavelength particles, acoustic streaming‐induced drag can further reduce trapping resolution by displacing particles away from pressure nodes.

#### 2.2.2 Acoustic Streaming

Acoustic streaming refers to steady fluid flow generated by viscous dissipation of acoustic energy. While ideal fluids exhibit zero net particle displacement under oscillatory motion, viscosity breaks time‐reversal symmetry, producing non‐zero mean flow. According to the dominant dissipation mechanism, acoustic streaming is broadly classified into boundary‐driven and bulk‐driven streaming [25, 60]. Boundary‐driven streaming originates from viscous shear within the Stokes boundary layer adjacent to solid‐fluid interfaces, producing localized recirculating flows known as Schlichting streaming (or inner streaming) (Figure 4A(i)) [61]. The slip velocity generated at the outer edge of the boundary layer, described by Rayleigh's streaming theory [62], drives a large‐scale recirculating flow in the fluid bulk, referred to as Rayleigh streaming (or outer streaming) (Figure 4A(ii)) [63, 64]. Rayleigh streaming therefore represents the bulk manifestation of boundary‐layer streaming imposed by the no‐slip boundary condition.

**FIGURE 4:**
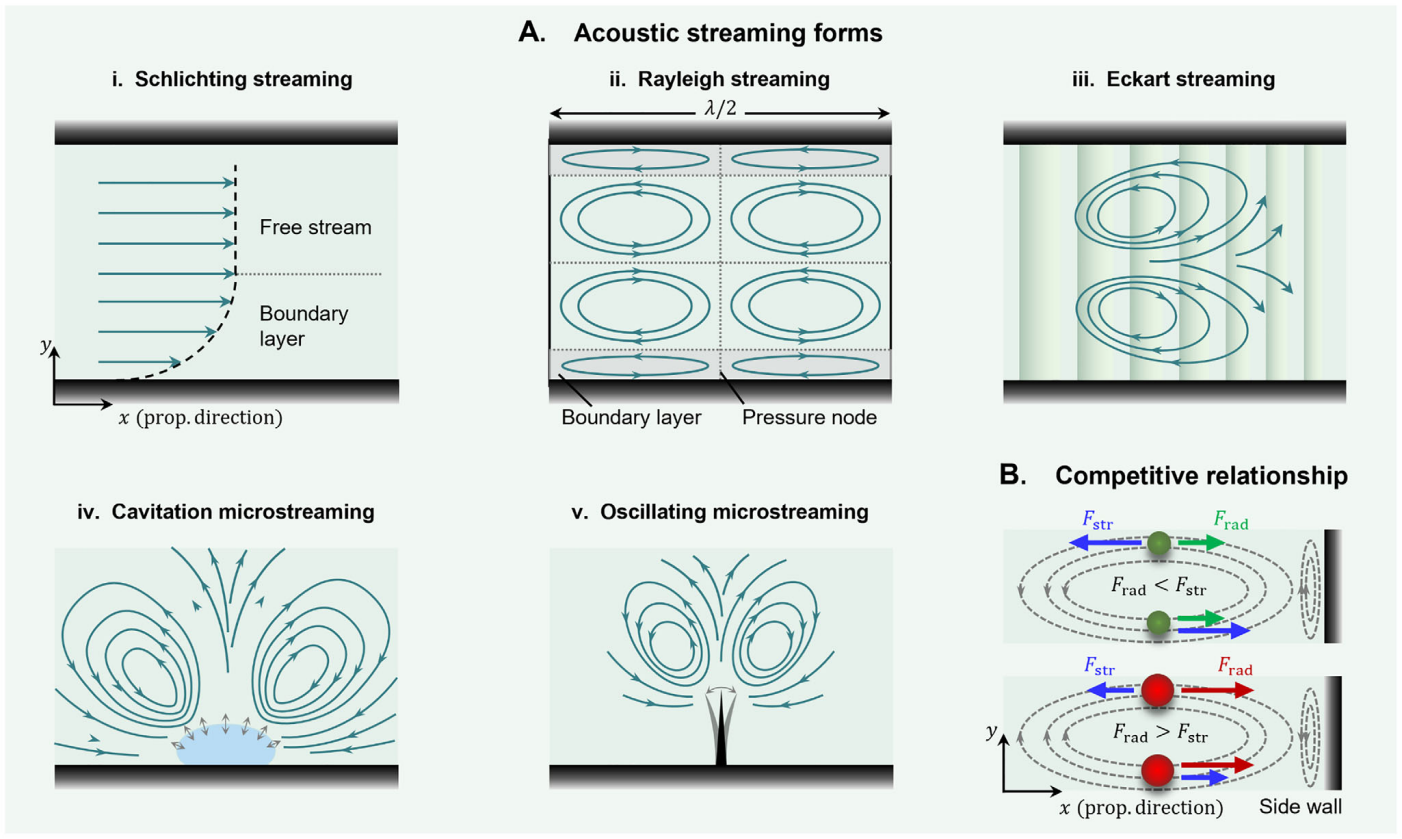
(A) Illustrations of the different forms of acoustic streaming. (i) The velocity gradient is normal to the boundary, and the velocity falls from its free‐stream value to 0. (ii) A system of Schlichting streaming vortices within the viscous boundary layer and Rayleigh streaming vortices in a channel with a standing wave propagating along *x*. (iii) A typical Eckart streaming flow including a backflow that arises due to the confined region. (iv) Symmetric acoustic streaming vortices generated by an oscillating microbubble. (v) Acoustic streaming generated by oscillating sharp structures. (B) The schematic demonstrates the radiation force and the sidewall‐induced streaming drag that act on different‐sized particles. Small particles follow sidewall‐induced streaming. Large particles migrate to the channel sidewall by the radiation force. For a particle with a radius *a* <  *a*_c_, the acoustic streaming force dominates. For a particle with a radius *a* >  *a*_c_, the acoustic radiation force dominates.

In contrast, bulk‐driven streaming results from acoustic energy attenuation within the fluid. In classical Eckart streaming, viscous attenuation alters the phase relationship between density and velocity oscillations, producing a non‐zero time‐averaged momentum flux along the acoustic propagation direction (Figure 4A(iii)) [65]. Gas–liquid interfaces, particularly oscillating microbubbles, can dramatically amplify streaming (Figure 4A(iv)) [66]. Cavitation microstreaming generated by resonant bubble oscillations produces localized, high‐velocity vortices that may exceed solid‐boundary streaming by orders of magnitude. Asymmetric oscillations further generate net hydrodynamic forces, forming the basis for bubble propulsion. Similarly, sharp edges and high‐aspect‐ratio microstructures enhance streaming by concentrating viscous dissipation within localized boundary layers (Figure 4A(v)).

Acoustic streaming can also be classified according to its intensity relative to the acoustic particle velocity. In Nyborg's classical perturbation theory [67], the streaming velocity *U*_str_ is assumed to be much smaller than the acoustic particle velocity *u*_1_. This slow‐streaming (creep‐flow) regime is characterized by a low streaming Reynolds number (*Re*_str_ =  ρ*U*_str_ *L*/µ  ≪  1, where *L* is the characteristic length and µ is the dynamic viscosity), such that convective inertia is negligible. At higher acoustic intensities, when *U*_str_ becomes comparable to *u*_1_ (*Re*_str_  ≥  1), the flow transitions to the fast‐streaming regime, in which the convective acceleration term (*u* · ∇ )*u* can no longer be neglected. This regime requires non‐perturbative analyzes, such as those developed by Zarembo [68, 69]. Importantly, the slow/fast classification is independent of the spatial classification (Schlichting, Rayleigh, or Eckart), and any streaming mechanism may occur in either regime depending on the acoustic intensity and system geometry. At sufficiently high acoustic amplitudes, nonlinear wave propagation becomes significant. This transition is characterized by an acoustic Reynolds number, $Re_{\mathrm{ac}}=\sigma \upsilon_{0}/(\omega \beta )\;>\;1$, where σ is the nonlinear coefficient, $\upsilon_{0}$ is the acoustic particle velocity amplitude, and β is the acoustic diffusivity. Under these conditions, waveform distortion, shock formation, and harmonic generation fundamentally alter both the magnitude and spatial distribution of acoustic streaming. Accurate analysis therefore requires coupling finite‐amplitude wave propagation (e.g., via Burgers’ equation) with inertial fluid dynamics [70].

Different Reynolds numbers characterize distinct aspects of acoustofluidic phenomena [28, 60, 71]. The oscillatory Reynolds number, *Re*_osc_  =  (*a*/δ)^2^, where δ denotes the viscous boundary layer thickness, governs the transition between radiation‐force‐dominated and streaming‐dominated particle manipulation. The streaming Reynolds number, *Re*_str_, characterizes the global streaming regime and is typically much smaller than unity in microfluidic systems, resulting in stable laminar flow. The interfacial Reynolds number, *Re*_int_, describes the localized microstreaming intensity near oscillating bubbles and is particularly relevant to cavitation‐driven propulsion (Section 4.2). The particle Reynolds number, *Re*_p_  =  ρ*U*_rel_ *d*_p_/µ, where *U*_rel_ is the relative particle velocity, determines the applicability of Stokes’ drag law. For spherical particles at low particle Reynolds numbers (*Re*_p_  ≪  1) and sufficiently far from boundaries, the streaming‐induced drag force is described by Stokes’ law [72]

(10) $$F_{\mathrm{str}}=-6\pi \mu aU_{\mathrm{rel}}$$

Because radiation forces scale with particle volume while streaming drag scales with particle radius, radiation dominates for larger particles, whereas streaming governs smaller ones (Figure 4B). The balance between these forces defines a critical radius *a*_c_ [73, 74] that determines manipulation regimes (Section 3).

### 2.3 Acoustic Devices and Sound Field Architectures

The functionality of acoustic manipulation systems relies on generating prescribed acoustic field distributions using appropriately designed devices. This section systematically reviews the common configurations of piezoelectric transducers and external acoustic actuation components employed in existing acoustic systems.

#### 2.3.1 Piezoelectric Transducer Configurations

Acoustic devices can be broadly classified into bulk acoustic wave and surface acoustic wave systems, based on the type of waves generated.

BAW devices typically operate at relatively low frequencies (<10 MHz) and provide greater penetration depth, making them suitable for large‐scale manipulation under low‐Reynolds‐number conditions. Unfocused traveling BAWs, commonly generated by planar transducers, propagate along the vibration direction (Figure 5A(i)). In contrast, focused BAW beams or acoustic vortices concentrate energy (Figure 5A(ii)), generating strong spatial gradient forces for applications such as acoustic tweezers.

**FIGURE 5:**
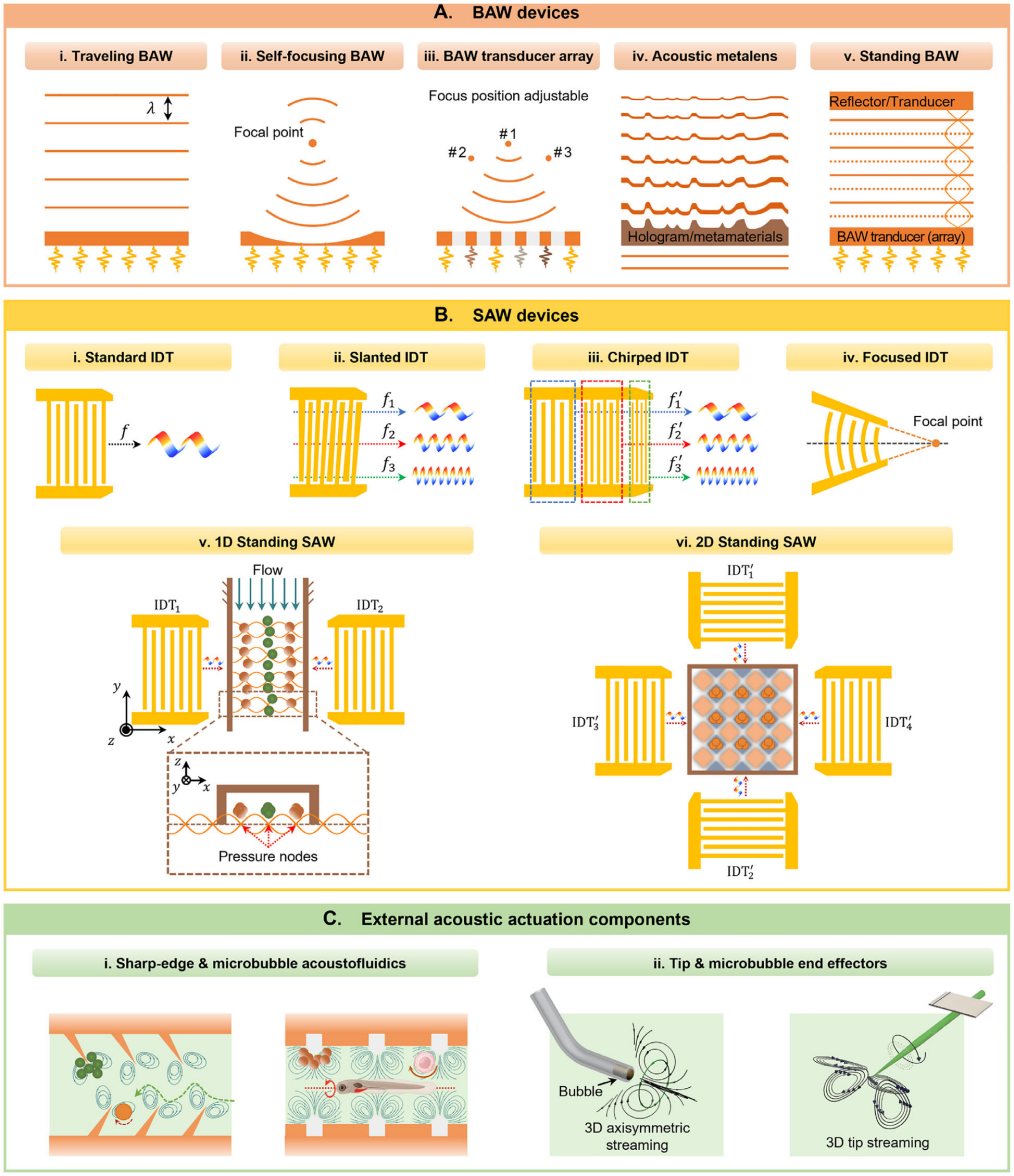
(A) BAW devices and acoustic field structures. (i) A piezoelectric transducer approximately produces a plane wave in a uniform medium. (ii) Self‐focusing acoustic beam generated by a concave transducer. (iii) Reconfigurable focused acoustic field generated by a multi‐element transducer array. (iv) The acoustic metalens placed in front of the path acoustic transmission modulates the acoustic wave to form customizable pressure distributions. (v) A resonator can be built using two opposing transducers or a combination of one transducer and one reflector. (B) Several common configurations of SAW transducer. (i) A standard interdigital transducer (IDT) can generate SAWs that propagate at a fixed wavelength. (ii) A tilted IDT can generate SAW beams of different frequencies along its finger length direction. (iii) A chirped IDT can achieve a wider bandwidth and gradually alter the SAW frequency by reducing or increasing the electrode spacing. (iv) A focused IDT can generate a strong and focused acoustic force or energy, which can be used to concentrate the acoustic energy to a focal point. (v) A linear standing SAW generated by a pair of opposite IDTs. (vi) A two‐dimensional standing SAW generated by two pairs of orthogonally arranged IDTs. (C) A schematic diagram depicts common external acoustic actuation components and the structures of their acoustic streaming. (i) The oscillating sharp edges and microbubbles can be utilized for single‐cell/organism manipulation and particle enrichment. (ii) Acoustofluidic end effectors based on bubble and tip, and their 3D acoustic streaming structure generated under acoustic field excitation. Reproduced with permission [108]. Copyright 2022, Springer Nature.

BAW focusing strategies include active phase control and passive phase modulation. Active approaches employ ultrasonic phased arrays composed of tens to hundreds of independently addressable elements. By electronically controlling the emission phase of each element, interference patterns can be tuned to generate one or multiple focal points with high flexibility (Figure 5A(iii)) [75, 76, 77, 78]. However, such systems require complex electronics and are limited by array size, leading to constraints in resolution and cost. Passive phase modulation relies on structured lenses or metasurfaces (acoustic metalenses) to tailor wavefronts (Figure 5A(iv)). Early designs used solid lenses with defined geometries (e.g., spherical or parabolic) [79, 80]. In contrast, acoustic metasurfaces introduce subwavelength structures, enabling precise wavefront shaping. This allows a single transducer to generate complex fields, such as vortices and holograms [81, 82]. For example, 3D‐printed acoustic holographic lenses can produce predefined patterns [83, 84], although dynamic reconfiguration remains challenging. Ongoing advances in fabrication and algorithm design are enabling increasingly reconfigurable acoustic metalenses. Over time, the dichotomy between static (holographic lens) and dynamic (phased arrays) approaches will gradually converge with the advent of reconfigurable metamaterials, rendering real‐time wavefront engineering without bulky electronic steering. Standing‐wave BAW devices are typically constructed using a single transducer with a reflector or a pair of opposing transducers (Figure 5A(v)) [85, 86, 87]. In acoustofluidic systems, traveling BAWs or SAWs can also be converted into standing waves within designed cavities via reflection or acoustic black hole structures [88, 89].

Traveling SAWs are generated by interdigital transducers (IDTs) and propagate along the substrate surface [90]. Their frequency, bandwidth, and directivity are determined by electrode geometry. By tailoring electrode patterns, complex acoustic fields can be produced (Figure 5B) [91, 92]. For example, slanted IDTs generate frequency‐graded beams across the aperture (Figure 5B(ii)) [93], while chirped IDTs enable broadband excitation by varying finger spacing along the propagation direction (Figure 5B(iii)) [94]. Focused IDTs, composed of concentric circular electrodes, create localized energy foci (Figure 5B(iv)) [95, 96, 97]. Flexible electrode designs and the integration of multiple IDTs enable diverse and even dynamically reconfigurable acoustic fields [98]. Traveling SAWs can also form standing waves within microchannel cavities [99, 100, 101]. However, due to structural constraints, standing SAW devices typically employ one or more opposing IDT pairs (Figure 5B(v, vi)) [102, 103].

#### 2.3.2 External Acoustic Actuation Components

External acoustic actuation components are acoustically responsive structures introduced into a system, such as sharp edges [104, 105], micropillars [106], or bubbles (Figure 5C) [107]. Under acoustic excitation, these features oscillate at the solid–liquid or gas–liquid interfaces, producing localized pressure fields and high‐velocity vortical streaming in the near‐field region. This acoustofluidic approach offers key advantages, including low excitation thresholds, strong localization, programmability, and ease of integration. It has demonstrated strong performance in applications such as microfluidic mixing, valveless pumping, single‐cell analysis, patterned assembly, and particle enrichment [33, 108].

The preceding subsections survey diverse acoustic device architectures, each characterized by distinct frequency regimes, penetration depths, spatial resolutions, and operational constraints. To facilitate cross‐platform comparison and guide device selection for specific manipulation tasks, Table 2 summarizes the key physical characteristics and practical limitations of the primary device classes discussed above.

**TABLE 2:** Comparison of acoustic device architectures.

Device type	Typical frequency	Penetration depth ^a^	Spatial resolution ^b^	Power/Intensity	Primary limitations
BAW (focused ultrasound) [109, 110]	40kHz to 10 MHz	Deep	850mm in air; Sub‐mm–mm	High	System complexity and cost
BAW (traveling wave) [111, 112, 113]	<10 MHz	Deep (cm)	∼λ/2 (Sub‐mm)	Moderate	Resolution fundamentally constrained by diffraction limit
BAW (standing wave) [114, 115, 116]	2–20 MHz	Moderate	∼λ/2 (37–370 µm)	Moderate	Fixed field patterns; relies strictly on cavity resonance
Acoustic holograms/ metasurfaces [82, 117]	0.5–5 MHz	Moderate	∼λ/2 (Sub‐mm)	Moderate	Static in most designs; slow dynamic reconfiguration
SAW (traveling wave) [118, 119, 120]	10 MHz to 1 GHz	Shallow (µm–mm)	∼λ/2 (2–200 µm in LiNbO_3_)	Low to moderate	Shallow penetration; coupling efficiency dependent
SAW (standing wave) [94, 121, 122]	10 MHz to sub‐GHz	Shallow	∼λ/2 (2–200 µm in LiNbO_3_)	Low to moderate	Periodic patterns inherently fixed
GHz resonators [123, 124, 125]	>1 GHz	Very shallow (µm)	Sub‐µm–µm	High (localized)	Extremely short working distance; thermal effects
Bubble/sharp‐edge streaming devices [104, 105, 126, 127]	5 kHz to MHz	Localized	Sub‐µm–µm	Low	Structure‐dependent; strong localization limits range
End effectors [128, 129, 130]	10 kHz to MHz	Localized	Sub‐µm–µm	Low	Proximity‐dependent; limited to near‐field operation

^a^ Penetration depth provides a qualitative indicator based on the device physics detailed in Section 2.3; quantitative values vary with media properties and operating conditions.

^b^ Spatial resolution is estimated from the diffraction limit (∼λ/2) in water (sound speed ∼1,482 m/s) at representative frequencies.

## 3 Particle Manipulation

Acoustic manipulation has emerged as a non‐contact, non‐destructive tool for precise control of droplets, bubbles, granules, and cells. Across gaseous and liquid environments, it enables regulation of particle motion, distribution, morphology, and even reaction behavior, primarily through acoustic radiation forces and acoustic streaming. These capabilities provide powerful approaches for fundamental studies and hold strong potential for applications in microfluidics, precision medicine, and smart materials.

A salient feature across these diverse applications is the broad spectrum of target objects, ranging from nanoscale exosomes and protein aggregates, to micron‐scale mammalian cells and bacterial spores, and further to millimeter‐scale droplets and multicellular organisms [28]. Despite their diverse physical properties (size, density, compressibility), particle size is the primary determinant of the acoustic response (Section 2.2), governing equilibrium positions, migration trajectories, and trapping stability. Specifically, the acoustic contrast factor ϕ and critical radius *a*_c_ define three distinct operational regimes according to *a* relative to δ and λ.

For submicron particles (*a*  ≤  δ), streaming‐induced Stokes drag (*F*_str_ ∝ *a*) dominates over acoustic radiation forces, necessitating high‐frequency (>100 MHz) resonators or sharp‐edge oscillators to generate strong localized microstreaming. In the intermediate regime (δ  ≪  *a*  ≪  λ), the Gor'kov radiation potential scales with *a* ^3^, making the acoustic radiation force *F*_rad_ the dominant mechanism for precise trapping, alignment, and patterning. For millimeter‐scale objects (*a*  ∼  λ), gravitational and higher‐order scattering forces become increasingly important, favoring airborne levitation and dynamic manipulation via phased arrays. Beyond determining the transition between acoustic radiation‐ and streaming‐dominated regimes, particle size also governs manipulation resolution and the scaling of secondary Bjerknes interactions. Table 3 summarizes these size‐dependent operational regimes, dominant physical mechanisms, representative device architectures, and typical applications.

**TABLE 3:** Size‐dependent operational regimes, dominant physical mechanisms, representative acoustic device strategies, and key applications in acoustic particle manipulation.

Size scale	Particle/Object examples	Dominant physical mechanism	Representative device/Strategy	Key applications
< 500 nm	Exosomes, viruses, protein aggregates, nanobubbles, synthetic nanoparticles	Acoustic streaming drag dominates (*F*_str_ ∝ *a*)	GHz BAW resonators, focused SAW with vortex streaming, sharp‐edge/microbubble array	Enrichment: Exosome isolation [131]; acoustic centrifugation [132, 133] Separation: Continuous particle aggregation [134]
0.5 ∼ 500 µm	Mammalian cells (e.g., HeLa), bacteria, *C. elegans* (dia. ∼50 –100 µm), zebrafish larvae (dia. ∼500 µm), cell clusters/spheroids	Acoustic radiation force dominates (*F*_rad_ ∝ *a* ^3^) for *a* ≫ δ	SAW (straight/focused/chirped IDTs, standing waves), BAW tweezers (phased arrays, focused transducers), acoustic end effectors, acoustic holographic lenses, Faraday‐wave platforms	Droplet steering: Transport, merging, and splitting [34, 135] Single‐particle Control: Trapping, translation, and rotation [81, 136, 137, 138, 139] Sieving: Cell sorting [114, 140, 141, 142]; antibody removal [143] Patterning: Cell pairing [144]; biological assembly [21, 22]; 3D tissue constructs [82, 145]
*>* 500 µm	Large droplets, entire organisms (e.g., zebrafish embryos ∼1–1.4 mm, *C. elegans* adults ∼1 mm), centimeter‐scale objects	Gravitational forces comparable to radiation forces	Single‐axis/multiaxis acoustic levitators, phased‐array tweezers; acoustic end effectors	Airborne levitation: Container‐free assembly [146]; volumetric display [109, 147, 148, 149, 150] Droplet handling: Translation and splitting [151] In vivo manipulation: Acoustic steering of glass spheres [152] Biomanipulation: Zebrafish embryo rotation and transport [108, 128, 138]

An application‐level performance comparison is also essential for guiding strategy selection before examining individual functional domains in detail. The application categories discussed in this section—including levitation, droplet manipulation, enrichment, separation, and patterning—differ substantially in target size range, evaluation criteria (e.g., capture efficiency, separation resolution, throughput, positioning accuracy, and pattern complexity), leading approaches, and key operational limitations. Table 4 consolidates these parameters across the five functional domains, providing an integrated comparative framework that complements the size‐dependent operational regimes established in Table 3.

**TABLE 4:** Comparison of application‐level performance across major acoustic manipulation functions.

Application class	Size range	Performance metrics	Optimal approach	Primary bottlenecks
Acoustic levitation	Sub‐mm to cm	Degrees of freedom, positioning precision, multi‐object capacity	Phased‐array holographic systems (highest reconfigurability and 3D control) [109, 150]	Limited throughput (limited to few objects); high system complexity and cost for large arrays
Droplet manipulation	nL to mL	Positioning precision, operation volume range, velocity	BAW phased arrays (spatial versatile) [34, 110]; SAW digital platforms (chip‐scale integration) [153]	Contact‐line pinning; evaporation for sub‐nanoliter droplets
Single‐particle manipulation	µm to mm	Degrees of freedom, spatial resolution, force sensitivity	Phased‐array/holographic tweezers (6‐DOF) [35]; planar SAW (rapid on‐chip operation) [154]; GHz resonators/ streaming end effectors (biocompatible localized manipulation) [125, 138]	Diffraction‐limited spatial resolution; lower force sensitivity relative to optical tweezers; complexity vs. integration trade‐offs
Enrichment	100 nm to µm	Capture efficiency, sample purity, processing speed	Bubble‐array/sharp‐edge streaming (∼93% exosome capture for in ∼3 min) [131]	Poor selectivity among similarly sized bioparticles; throughput constraints
Sieving (Separation)	100 nm to µm	Separation efficiency, volumetric throughput, resolution	Tilted‐angle standing surface acoustic wave (>95% separation efficiency for cells/microparticles) [140]	Trade‐off between resolution and flow rate; sensitivity to biofluid acoustic impedance variations
Patterning	µm to mm	Pattern fidelity, throughput, dynamic reconfigurability	Acoustic holography (arbitrary 3D patterns) [155, 156]; standing wave fields (high‐throughput periodic lattices) [157]	Slow dynamic reconfiguration (∼ seconds); fidelity vs. throughput trade‐offs; transient pattern stabilization

### 3.1 Acoustic Levitation

Acoustic levitation uses radiation forces generated by high‐intensity or high‐frequency sound waves to counteract gravity, enabling stable suspension of objects [147]. As a containerless technique, it allows flexible manipulation of samples with varying sizes and compositions [158, 159, 160]. For example, single‐axis levitation enables drying‐mediated nanoparticle self‐assembly in levitated droplets (Figure 6A) [146], eliminating boundary effects such as the coffee‐ring phenomenon. Levitated droplets can also undergo resonance‐driven shape transitions, forming highly stable bubble structures even without surfactants [161, 162, 163]. Multiaxis levitation systems provide enhanced force and stability [148, 164]. Ultrasonic phased‐array platforms enable coordinated manipulation of multiple droplets by dynamically shifting standing wave nodes, and can even control centimeter‐scale objects (Figure 6B) [149]. Holographic acoustic tweezers based on phased arrays allow real‐time reconfiguration of acoustic fields, supporting independent and fully 3D manipulation of multiple particles [165]. Integration with RGB illumination and haptic feedback further enables multimodal interaction (Figure 6C(i)) [150]. However, such systems may face limitations in biocompatibility and trapping precision. In contrast, single‐sided phased‐array systems offer larger workspaces and simpler architectures (Figure 6C(ii)) [109, 166]. By employing optimization algorithms, they can generate diverse trapping modes, including twin traps, vortex traps, and bottle‐shaped traps, enabling precise 3D manipulation without reflectors or lenses.

**FIGURE 6:**
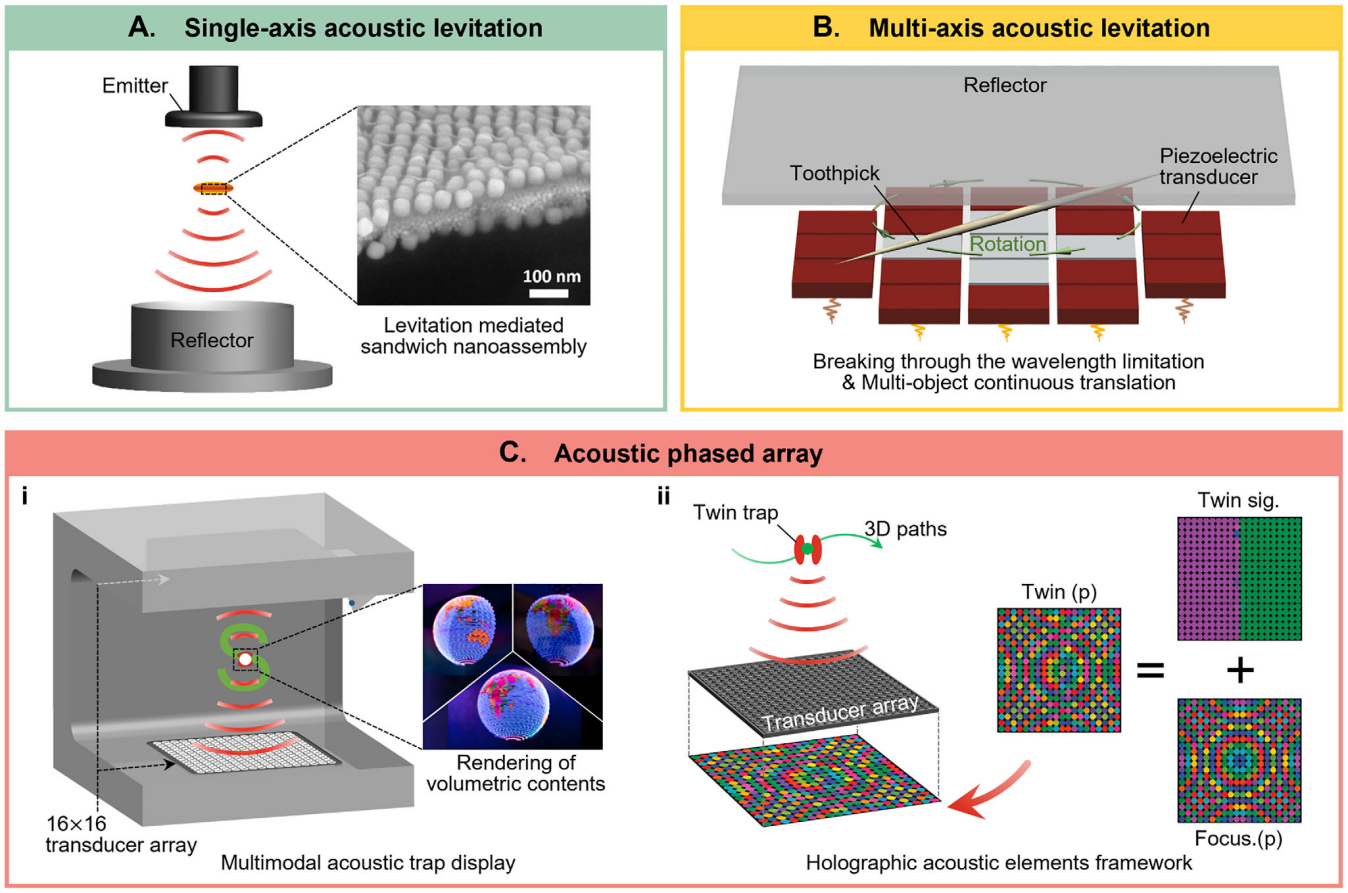
(A) Single‐axis acoustic levitation devices. Levitation‐mediated self‐assembly of a bilayer nanomembrane (inset). Reproduced with permission [146]. Copyright 2019, American Chemical Society. (B) Multiaxis acoustic levitation devices: contactless transport of an elongated object. Reproduced with permission [149]. Copyright 2013, National Academy of Sciences. (C) (i) A levitating volumetric display that can simultaneously deliver visual, auditory, and tactile content. 3D raster images show rich color information (inset). Reproduced with permission [150]. Copyright 2019, Springer Nature. (ii) The holographic acoustic elements framework based on a single‐sided phased‐array can generate acoustic structures shaped as tweezers, twisters, or bottles. The phase modulations of transducers for generating acoustic traps can be decomposed into focusing elements and holographic signatures. Reproduced with permission [109]. Copyright 2015, Springer Nature.

Overall, acoustic levitation provides a versatile platform for stable, contactless manipulation in complex environments. Phased‐array holographic systems offer the greatest flexibility for reconfigurable 3D manipulation, whereas single‐ and multiaxis standing‐wave systems remain the most robust for routine containerless processing. The principal bottleneck lies in the trade‐off between trapping stiffness and workspace size: increasing the number of transducer elements enhances spatial control but also substantially increases system complexity and cost. Throughput remains another major limitation, as most current systems manipulate only one or a few objects, whereas practical applications, such as high‐throughput chemical analysis and additive manufacturing, require massively parallel operation. From a mechanistic perspective, the coupling between multiple levitated objects and the surrounding medium under high‐intensity acoustic fields remains poorly understood, particularly the roles of thermal effects and nonlinear acoustic streaming in destabilizing multi‐object levitation. Future advances will likely rely on integrating real‐time feedback with adaptive wavefront control to enable autonomous responses to changing sample properties and environmental disturbances.

### 3.2 Droplet Manipulation on Surfaces

Precise droplet control is critical for microfluidics, biosensing, drug delivery, and lab‐on‐a‐chip systems. Conventional approaches often require complex surface treatments or external fields (optical, electrical, magnetic), which can limit biocompatibility and flexibility [167, 168]. Acoustic methods offer a non‐contact, label‐free alternative with improved adaptability [169, 170].

#### 3.2.1 BAW‐Driven Droplet Steering

BAW devices manipulate droplets via two main mechanisms: acoustic radiation force traps and asymmetric acoustic streaming. Focused phased arrays can generate twin‐trap fields to capture and steer droplets along predefined paths on superhydrophobic (Figure 7A) or slippery surfaces [34, 171, 172]. On superhydrophilic surfaces, strong pinning and viscous effects hinder droplet motion. To address this, tightly focused acoustic fields can induce droplet contraction (“acousto‐dewetting”), enabling controlled motion across a wide range of viscosities and wettabilities (Figure 7B) [110]. More recently, sound‐controlled fluid processors have been developed to allow multifunctional manipulation—including translation and splitting—over a broad range of droplet volumes (1 nL–3 mL) and surface tensions (17.9–72.0 mN m^−1^) (Figure 7C) [151]. Moreover, integration with multiple acoustic sources enables automated, enclosed lab‐on‐a‐chip operations.

**FIGURE 7:**
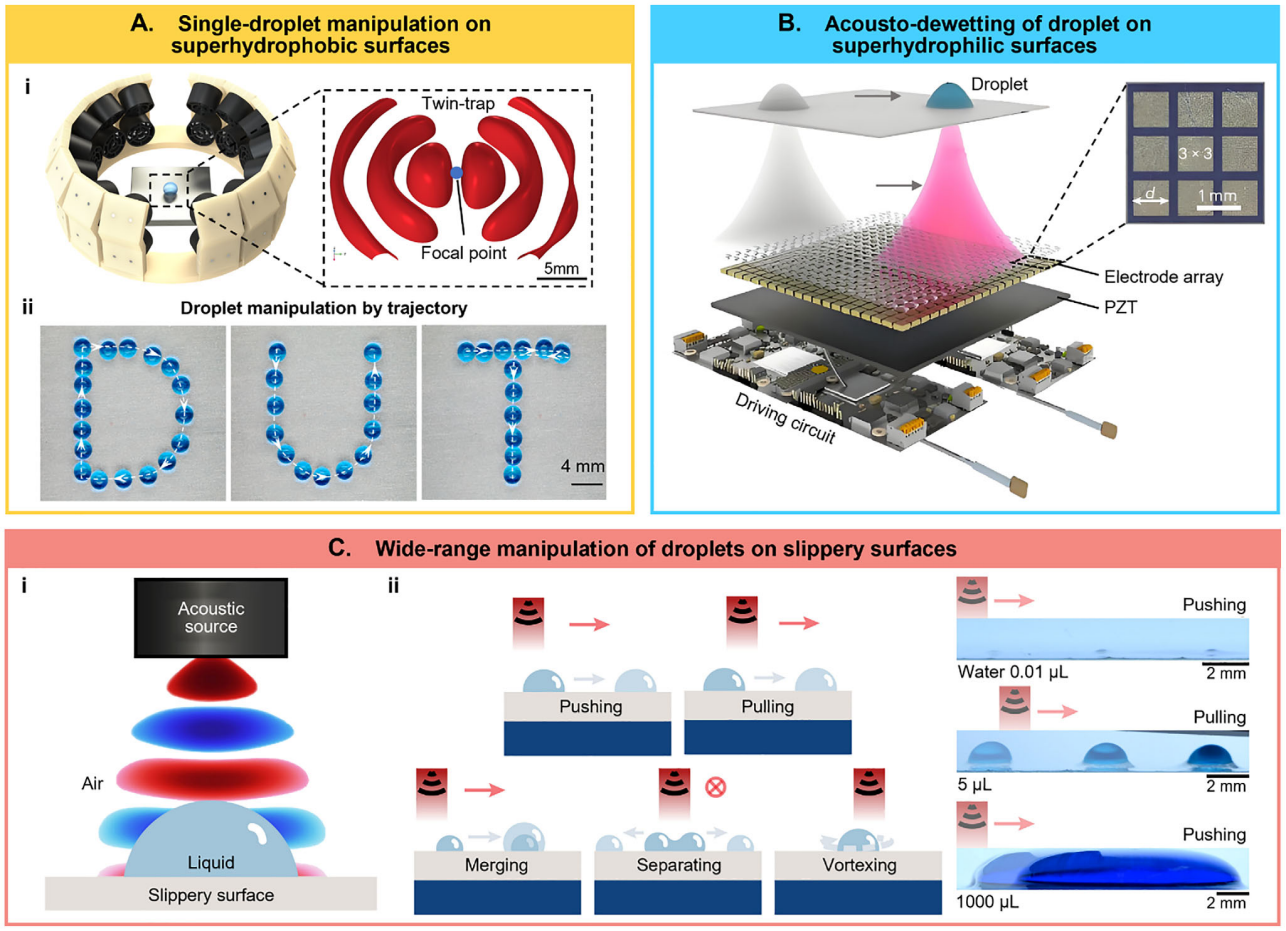
(A) (i) Schematic of the droplet ultrasonic tweezer and simulated shape of twin‐trap ultrasonic field. (ii) Stacked image of a controlled droplet moving along a predetermined trajectory as it follows the focal point of the twin‐trap acoustic field. Reproduced with permission [34]. Copyright 2023, AAAS. (B) Schematic illustrating the experimental setup, where the focused ultrasound beam can be electronically steered to spatiotemporally confine within the droplets. The inset shows an enlarged view of a 3 × 3 electrode array. Reproduced with permission [110]. Copyright 2025, Springer Nature. (C) (i) Schematic of the sound‐controlled fluid processor. The acoustic wave generated by the acoustic source in air drives droplet motion on a slippery surface. (ii) Schematics of droplet manipulation by the processor, including pushing, pulling, merging, separating, and vortexing, while showing acoustic manipulation of water droplets with different volumes. Reproduced with permission [151]. Copyright 2025, AAAS.

#### 3.2.2 SAW‐Driven Droplet Steering

SAW devices are well suited for chip‐scale fluid manipulation due to their compactness and integrability [173, 174]. By tailoring interdigital transducer (IDT) geometries, complex acoustic fields and streaming patterns can be generated. For example, orthogonal slanted IDTs create frequency‐tunable gradient fields for droplet trapping, transport, and centrifugation [175]. “Digital acoustofluidics” extends this concept to programmable, high‐throughput droplet operations, including transport, merging, mixing, and splitting via streaming vortices (Figure 8A) [135]. Advanced designs such as dual‐mode IDTs produce vortex‐driven transport over long distances (Figure 8B) [153], while integrated sensing systems enable closed‐loop control of droplet position and behavior [176]. These developments significantly enhance automation and precision in on‐chip fluid handling.

**FIGURE 8:**
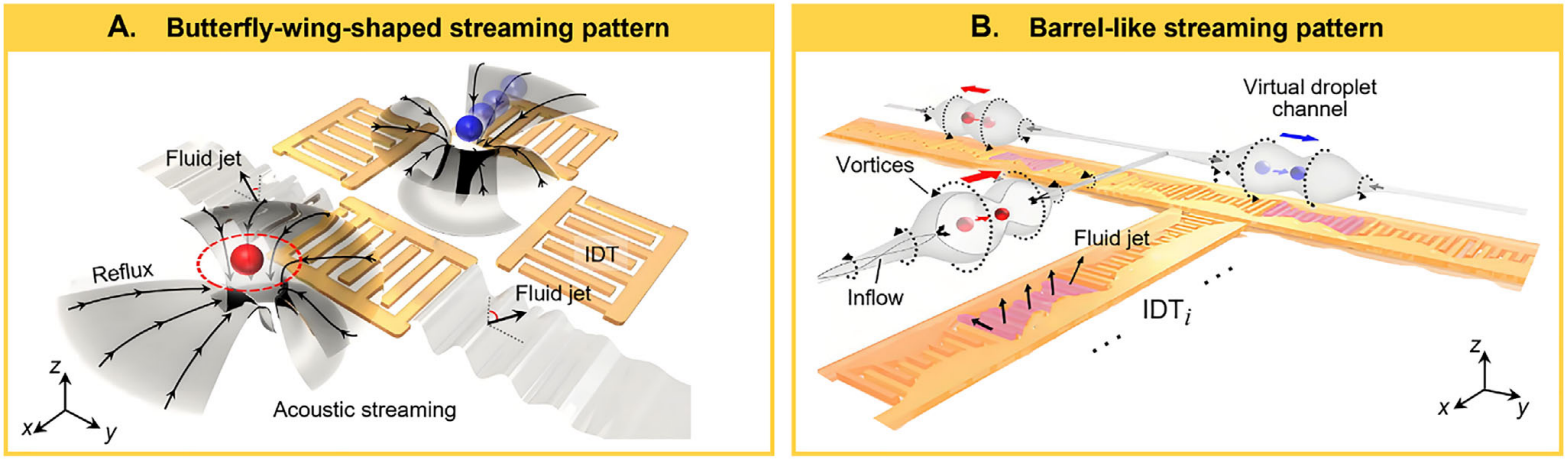
(A) Schematic showing one unit consisting of four IDTs in the digital acoustofluidic device. The droplets are separately trapped at the two symmetric hydrodynamic wells near the flanks of an IDT. Reproduced with permission [135]. Copyright 2018, Springer Nature. (B) Schematic showing a typical droplet processing unit. The droplets (i.e., red and blue spheres) over the transducers are guided into the center between the barrel‐like vortices. These droplets are unidirectionally routed along the linear array of IDTs by shifting the sequence of working frequencies. Reproduced with permission [153]. Copyright 2020, AAAS.

For surface droplet manipulation, BAW‐based phased‐array tweezers provide greater spatial versatility across a wide range of viscosities and surface wettabilities, whereas SAW‐based devices offer superior chip‐scale integration and programmable, high‐throughput operation. However, contact‐line pinning remains a persistent limitation for both approaches, fundamentally constraining positioning accuracy and reproducibility, particularly for sub‐nanoliter droplets on conventional substrates [177]. From a mechanistic perspective, the coupling between internal acoustic streaming and moving contact lines under non‐equilibrium wetting conditions remains poorly understood, and a predictive framework for triple‐line force balance under acoustic excitation is still lacking. Future progress will likely depend on integrating closed‐loop feedback control with advanced surface engineering to achieve reliable, automated droplet manipulation across broader ranges of droplet volume, viscosity, and substrate chemistry.

### 3.3 Liquid‐Phase Acoustofluidics

Compared with gaseous media, liquid environments not only serve as the primary carrier for biological particles and cells but also provide richer physical mechanisms and broader application scenarios for acoustic manipulation. This section systematically reviews the operating principles of acoustic devices across various applications, categorized by the behavioral modes of manipulated particles.

#### 3.3.1 Single‐Particle Manipulation

*BAW Tweezers*: Single‐particle manipulation using acoustic tweezers typically relies on localized, tunable acoustic radiation force traps or acoustic streaming vortices [178, 179]. Phased array technology, with dynamic beam‐steering capability, shows strong potential for manipulating individual particles in complex environments [180]. For instance, a spherical phased‐array can stably trap a glass sphere within a pig bladder and guide it along a predefined trajectory (Figure 9A(i)) [152]. However, arrays composed of discrete transducer elements are still limited in spatial resolution and are prone to beam defocusing in inhomogeneous media [181, 182]. To address these challenges, Yang et al. developed a self‐navigating 3D acoustic tweezer system that integrates real‐time ultrasonic imaging with a feedback loop, enabling reconstruction and maintenance of high‐precision acoustic focusing even in strongly scattering environments (Figure 9A(ii)) [136]. Future developments are expected to integrate intelligent control algorithms with real‐time imaging techniques to achieve robust micromanipulation in heterogeneous media.

**FIGURE 9:**
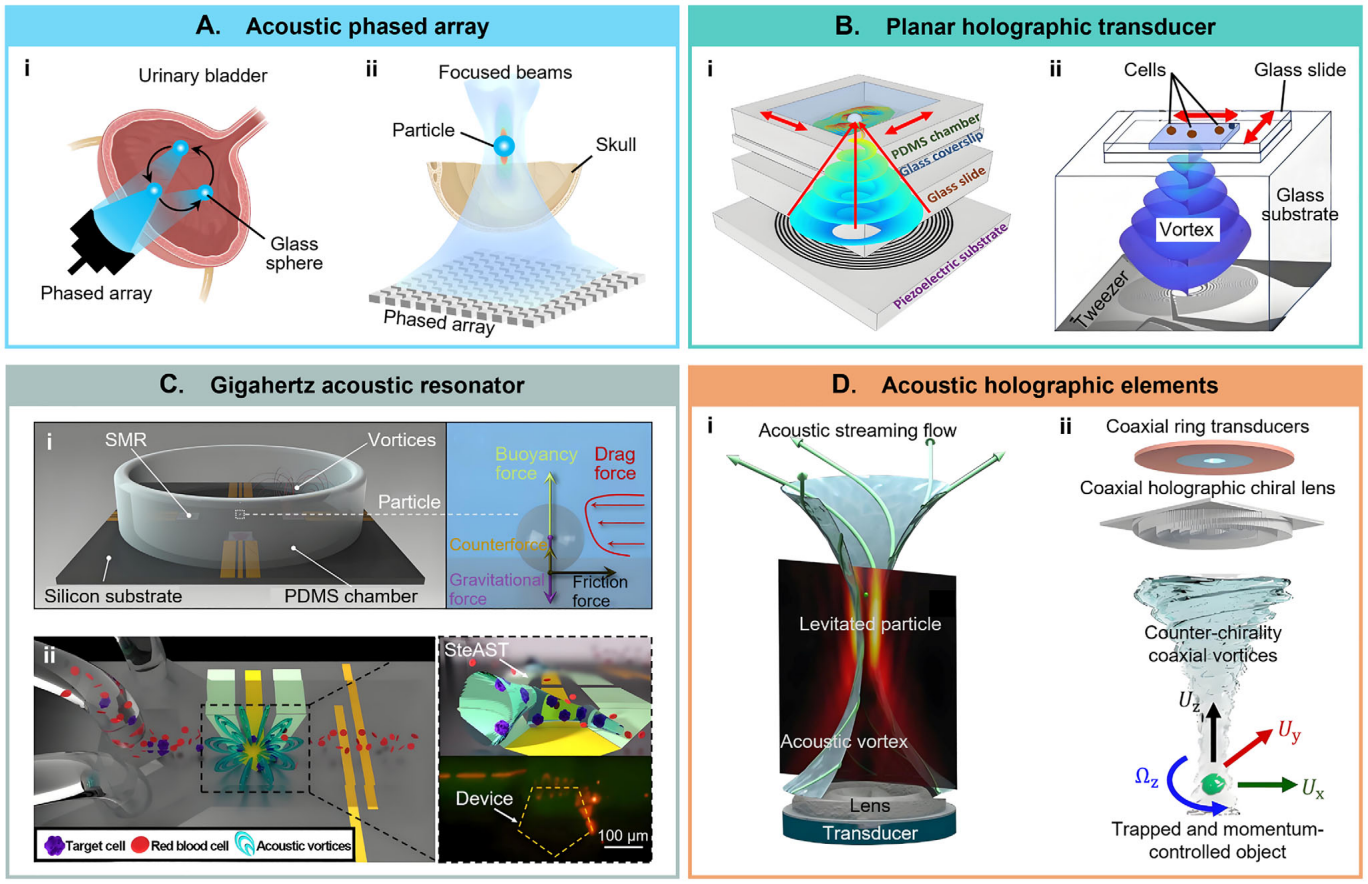
(A) (i) Acoustic manipulation of a glass sphere in a pig bladder. Phased‑array systems achieve deep‑tissue trapping, demonstrating that acoustic manipulation can operate in highly scattering biological media. Reproduced with permission [152]. Copyright 2020, National Academy of Sciences. (ii) Schematic of time reversal‐based acoustic tweezers through an ex vivo human skull. Reproduced with permission [136]. Copyright 2021, AAAS. (B) (i) Scheme illustrating the composition of the Archimedes‐Fermat acoustical tweezers. Planar holographic transducers fold a 3D focused vortex onto a flat chip, illustrating a route to miniaturization. Reproduced with permission [137]. Copyright 2019, AAAS. (ii) Synthesis of a spherically focused acoustical vortex by spiraling active electrodes. A cell located at the center of an acoustic vortex is selectively trapped and manipulated. Reproduced with permission [185]. Copyright 2020, Springer Nature. (C) (i) Diagram of the GHz BAW‐based single‐particle manipulation platform and schematic of the forces related to the manipulation of a single particle. Reproduced with permission [125]. Copyright 2022, American Chemical Society. (ii) A schematic of the stereo acoustic streaming (SteAST) platform. The detailed images show a cartoon profile of SteAST and the stacked image that demonstrates the trajectory of a fluorescent particle in the tunnel. Reproduced with permission [187]. Copyright 2022, Springer Nature. (D) (i) Focused acoustic vortex for in‐plane particle capture. Reproduced with permission [81]. Copyright 2021, Springer Nature. (ii) Schematic of a chirality‐tunable acoustic vortex tweezing device, which can generate a chirality‐tunable acoustic vortex beam to trap an object and control the object's rotation. Reproduced with permission [35]. Copyright 2024, AAAS.

To enable miniaturization and high‐precision manipulation, planar, microfabrication‐compatible architectures have been developed for acoustic field modulation, facilitating flexible on‐chip manipulation. Early designs using spiral IDTs generated spiral acoustic fields but suffered from weak focusing, high side lobes, and unstable 3D trapping [183, 184]. The folded acoustic vortex approach overcomes these limitations by implementing Archimedes‐Fermat spiral electrodes on a planar piezoelectric substrate (Figure 9B(i)), enabling efficient 3D focusing with reduced side‐lobe intensity [137]. With improved thermal management, planar holographic acoustic tweezers can achieve highly selective, non‐destructive manipulation of single living cells (Figure 9B(ii)) [185].

At gigahertz (GHz) excitation frequencies, planar acoustic devices generate highly localized fluid motion [123, 124, 186]. This localization results from the frequency‐dependent viscous attenuation length $\delta_{\mathrm{att}}\;∼\;1/\alpha$, which scales as ω^−2^ in the viscous regime (Section 2.1), confining the acoustic field to the immediate vicinity of the transducer surface. A single resonator produces petal‐shaped vortices at its periphery (Figure 9C(i)), while multi‐resonator arrays enable linear superposition of vortex fields, allowing programmable particle trajectories [125]. In addition, stereo acoustic streaming (SteAST) from a single resonator can create a “virtual tunnel” for selective capture, controlled release, and isolation of cells, including tumor cells from blood samples (Figure 9C(ii)) [187]. Although planar piezoelectric transducers offer advantages in integration, biocompatibility, and response speed, their performance is still constrained by limited operating range, thermal effects, and scalability.

Acoustic holographic lenses, as passive field‐control devices, shape acoustic wavefronts via engineered structures to produce predefined 3D field distribution. For instance, integrating a structured polydimethylsiloxane (PDMS) holographic lens with a single transducer enables hybrid 3D single‐beam acoustic tweezer (Figure 9D(i)) [81]. A coaxial holographic chiral lens further enables independent modulation of high‐ and low‐frequency waves into counter‐rotating vortex beams (Figure 9D(ii)), allowing precise control over the chirality and orbital angular momentum of trapped objects, and thus their rotation direction and speed [35]. Collectively, these approaches—including localized traps, streaming vortices, and holographic fields—enable precise single‐particle manipulation across media ranging from homogeneous fluids to opaque biological tissues, significantly expanding the applicability of acoustofluidics.

*SAW Tweezers*: SAW‐based acoustic tweezers have been widely used for single‐particle manipulation in microfluidic chambers or sessile droplets [94, 122, 188, 189]. However, achieving simultaneously multi‐degree‐of‐freedom (multi‐DOF) translational and rotation remains challenging. To overcome this limitation, Shen et al. developed a joint subarray acoustic tweezer system, enabling six‐DOF manipulation of single cells [154]. The system employs three sets of IDT arrays (outer, middle, and inner), each operating at distinct frequencies (Figure 10A). Selective activation of subarrays allows independent control of acoustic radiation forces and streaming vortices, enabling decoupled regulation of cell translation, rotation, and deformation (Figure 10B). Future progress is expected to leverage multiphysics coupling to achieve highly flexible and precise manipulation.

**FIGURE 10:**
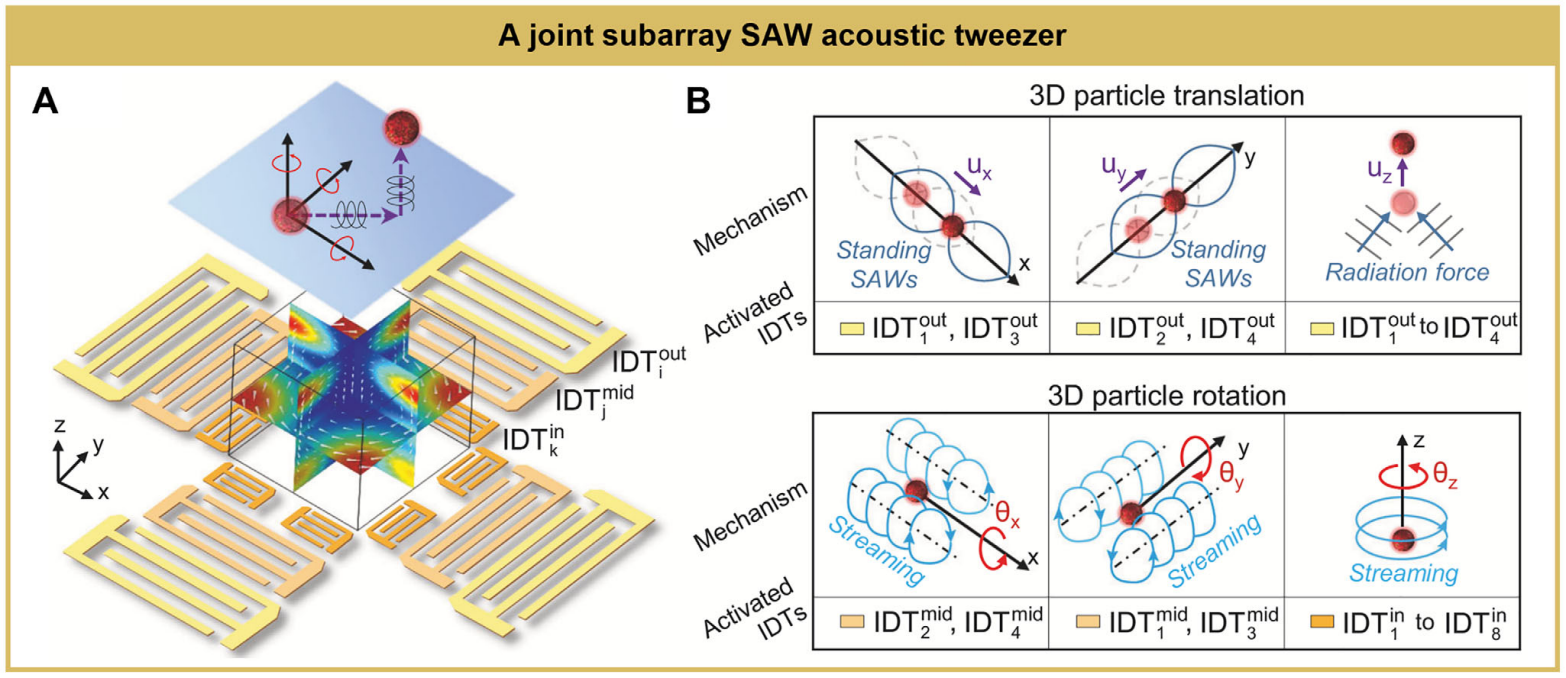
(A) A schematic of a joint subarray acoustic tweezer chip. This IDT array configuration generates controllable acoustic pressure fields and streaming patterns, allowing precise control of the multi‐DOF motions of single cells. (B) Schematics illustrating the mechanism of this acoustic tweezer system for six fundamental single‐cell manipulations. Reproduced with permission [154]. Copyright 2024, Springer Nature.

*Acoustic Streaming Tweezers Formed by External Components*: In microfluidic systems, acoustically actuated structures—such as anchored bubbles [107, 126, 190], sharp edges [191, 192], and end effectors [129, 130, 193, 194, 195]—play key roles in single‐cell manipulation and analysis. For example, patterned chips with circumferential vibration can generate distinct micro‐vortex modes by tuning vibration direction and micropillar configuration, enabling non‐contact cell manipulation (Figure 11A(i)) [196]. Compared with microneedles, this approach reduces operator dependency and minimizes mechanical damage to cells. Furthermore, under acoustic excitation, oscillating anchored microbubbles in microchannels generate localized steady flows that can rotate single cells or cell clusters around arbitrary axes and even stepwise rotation of entire *C. elegans* (Figure 11A(ii)) [127]. When integrated with micro‐force sensors, these systems further enable 3D mechanical characterization of biological specimens, including pollen tube cells and nematodes [197].

**FIGURE 11:**
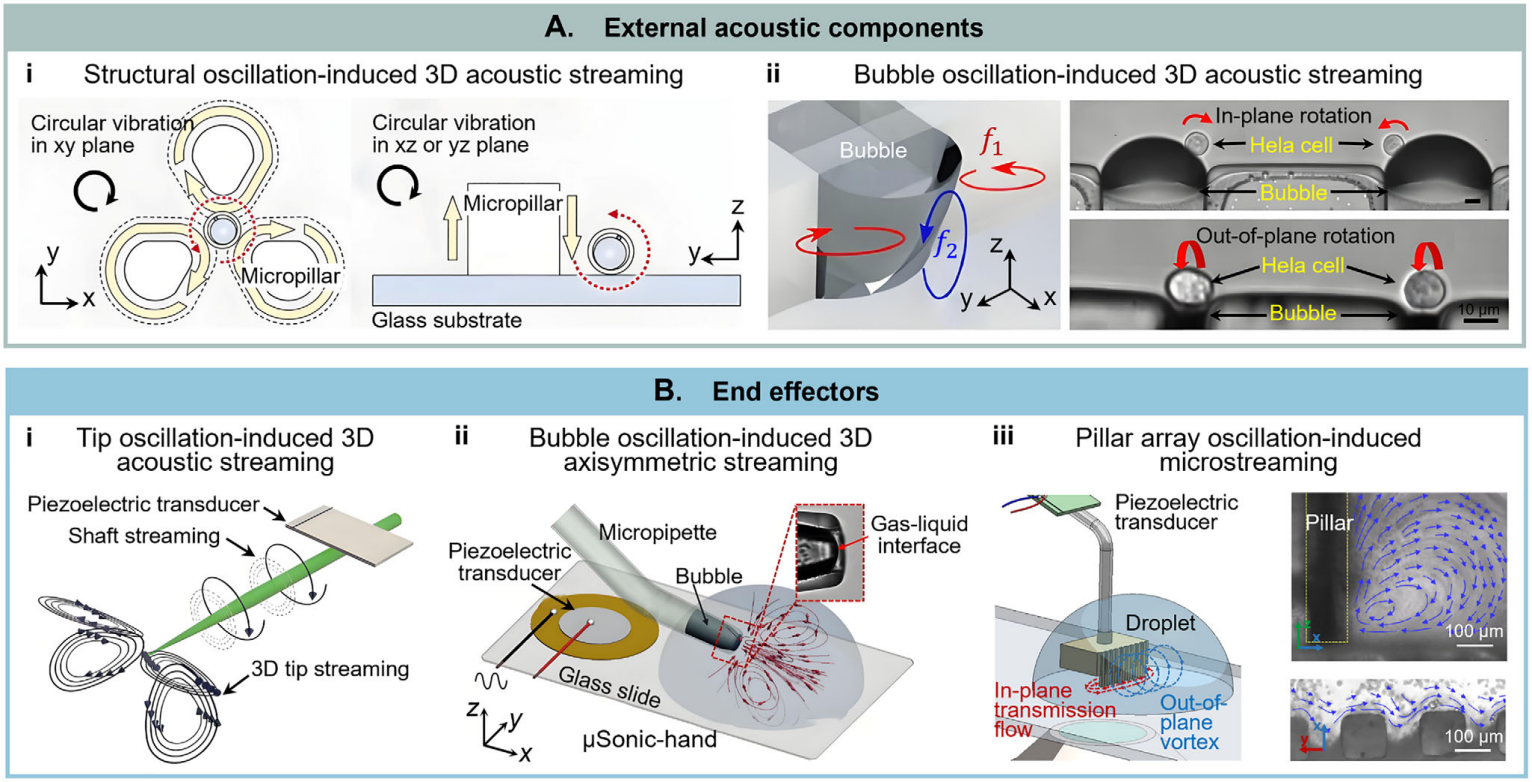
(A) (i) Cell rotation based on vibration‐induced flow. Reproduced with permission [196]. Copyright 2015, Springer Nature. (ii) 3D sketch demonstrating in‐plane and out‐of‐plane acoustic microstreaming vortices (left). High‐speed imaging showing in‐plane and out‐of‐plane rotation of HeLa cells (right). Reproduced with permission [127]. Copyright 2016, Springer Nature. (B) (i) A capillary tip‐based end effector with 3D acoustic streaming induced by tip oscillation. Reproduced with permission [108]. Copyright 2022, Springer Nature. (ii) Bubble‐based end effector: the gas–liquid interface oscillates in response to the acoustic waves emitted by the piezoelectric transducer, generating 3d axisymmetric streaming. Reproduced with permission [138]. Copyright 2025, AAAS. (iii) Micromanipulation using the acoustohydrodynamic pillar array as an end effector (left). Out‐of‐plane vortex near the single pillar and in‐plane transmission flow surrounding the whole pillar array (right). Reproduced with permission [139]. Copyright 2025, National Academy of Sciences.

The introduction of end effectors represents a key advance in acoustic manipulation, shifting from localized actuation to selective, spatially flexible control of particles, thereby enhancing system versatility and autonomy. For instance, integrating a capillary transducer assembly with a robotic arm yields a microfluidic platform that combines spatial agility with high‐throughput automation (Figure 11B(i)) [108]. A bubble‐based soft acoustic micro‐gripper enables non‐destructive capture, transport, and both in‐plane and out‐of‐plane rotation of objects ranging from 20 µm beads to 1.4 mm zebrafish embryos. This approach has been validated across diverse biological samples—from 7 µm yeast cells to 1 mm zebrafish embryos [115, 118, 198, 199]—demonstrating its versatility as a cross‐scale biomanipulation platform (Figure 11B(ii)) [128, 138]. Recent advances employing comb‐like acoustic streaming end effectors further enable complex, dynamic manipulation of droplets, single cells, and *C. elegans* through frequency‐dependent microflow patterning (Figure 11B(iii)) [139]. Future developments will focus on deformable micropillars and reconfigurable array structures to further expand the fluidic manipulation capabilities. Overall, tip‐ and bubble‐based end effectors combine ease of use, flexibility, and precision, offering powerful tools for life science research, precision medicine, and microscale synthesis. It should be cautioned, however, that intense streaming vortices may exert significant shear stress, which, although beneficial for mixing, could compromise membrane integrity in delicate primary cells.

In summary, single‐particle manipulation encompasses a wide range of acoustic platforms, each with distinct advantages and limitations. Phased‐array and holographic tweezers provide the greatest dexterity, enabling up to six degrees of freedom, but their high cost and system complexity limit widespread adoption. Planar SAW devices offer rapid, integrated on‐chip operation but remain limited in achieving simultaneous multi‐degree‐of‐freedom control. GHz resonators and streaming‐based end effectors excel in localized, biocompatible manipulation, although their trapping range and force output are inherently constrained. Across all platforms, the acoustic diffraction limit (∼λ/2) imposes a fundamental bound on spatial resolution, preventing subcellular targeting. From a mechanistic perspective, the dynamic interplay between acoustic radiation force and streaming‐induced drag near deformable objects remains poorly understood. In addition, the absence of standardized metrics for quantifying trapping stiffness and force precision continues to impede objective comparison among platforms and rational device selection for specific biological applications.

#### 3.3.2 Particle Enrichment

Particle enrichment is a critical preprocessing step in biomedical analysis, as its efficiency and selectivity directly determine the sensitivity and reliability of downstream assays [200, 201, 202]. Conventional methods, such as centrifugation and filtration, are often limited by complex workflows, contamination risks, and large sample requirements. In acoustofluidic systems, enrichment typically relies on two mechanisms: (i) selective trapping of target particles in acoustic potential wells or vortices, and (ii) centrifugal manipulation driven by acoustic streaming within droplets or fluid chambers.

*Acoustic potential wells or acoustic vortices*: Oscillating bubbles and sharp‐edge microstructures enable rapid enrichment of micro‐ and submicron particles by generating strong localized acoustic streaming and steep pressure gradients [203, 204, 205, 206]. Particles are transported toward the vibration source by hydrodynamic drag and subsequently trapped by acoustic radiation forces. Microbubble geometry plays a crucial role in shaping flow fields. Elliptical bubbles, for example, generate stable four‐vortex streaming patterns over a broad frequency range, with improved shear uniformity compared to spherical bubbles [207]. Similarly, sharper tip angles in microstructures significantly enhance streaming velocity and produce more complex vortex patterns, which can be precisely tuned via geometric design [208]. These approaches enable high‐throughput particle capture. For instance, a microbubble‐array‐based acoustofluidic trap achieves rapid (∼3 min) and high‐purity (93%) isolation of exosomes (∼88 nm) [209, 210] directly from whole blood (Figure 12A) [131]. A wedge‐structured integrated chip enables simultaneous capture of cells, bacteria, and vesicles from saliva, along with on‐chip virus lysis, RNA enrichment, and antibody purification (Figure 12B), achieving a 32‐fold increase in RNA detection sensitivity and antibody detection limits as low as 15.6 pg mL^−1^ [211].

**FIGURE 12:**
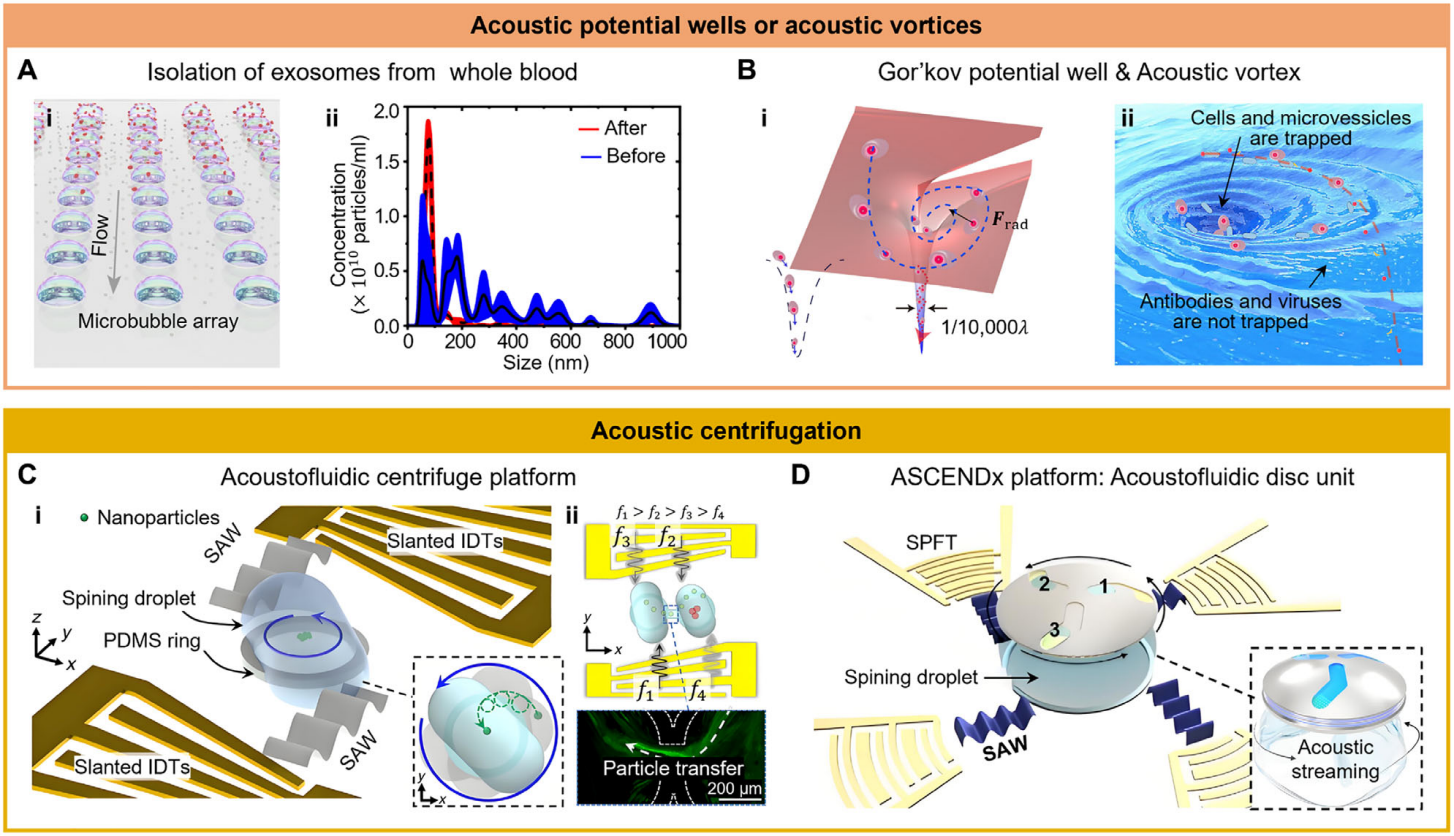
(A) (i) When a fluid containing different‐sized particles runs through the device, the oscillating microbubbles can trap the particles. (ii) The size distribution of the sample before and after treatment. Reproduced with permission [131]. Copyright 2025, AAAS. (B) (i) Visualization of the sub‐wavelength Gor'kov potential well near a wedge microstructure. (ii) Illustration depicting the selective acoustic trapping. Reproduced with permission [211]. Copyright 2025, AAAS. (C) (i) Operating mechanism of the acoustofluidic centrifuge platform: enrich nanoparticles in open microdroplets. (ii) Schematic of the dual‐droplet acoustofluidic centrifuge. Reproduced with permission [132]. Copyright 2021, AAAS. (D) Schematic of the acoustofluidic disc unit of the ASCENDx platform. As SAWs propagate into the droplet, a helical vortex is formed (inset), causing the droplet‐disc system to rotate. Reproduced with permission [133]. Copyright 2024, AAAS.

*Acoustic centrifugation*: Droplets serve as open, tunable microreactors for particle enrichment, offering low sample consumption, operational flexibility, and high efficiency [89, 212, 213]. By coupling acoustic streaming, secondary flows, and particle migration, integrated workflows for enrichment, encapsulation, and detection have been established [119]. For example, tilted IDTs drive particles along spiral trajectories toward the droplet center (Figure 12C) [132]. A dual‐droplet centrifugal system enables one‐step separation and collection of nanoparticles with size differences exceeding 1.5‐fold. The Acoustic Separation and Concentration of Exosomes and Nucleotide Detection (ASCENDx) system further integrates droplet‐based enrichment with molecular diagnostics (Figure 12D), streamlining analytical workflows and advancing exosome‐based diagnostics [133].

*Acoustic‐responsive targeted aggregation*: Acoustic techniques provide a non‐invasive strategy for targeted drug delivery with deep tissue penetration [12, 214, 215, 216]. Achieving in vivo targeted aggregation requires acoustically responsive carriers. One promising approach—acoustic field navigation via microbubble carriage—functionalizes drug carriers with phospholipid–polymer hybrid microbubbles (Figure 13A(i)) [217]. These gas microbubbles exhibit low acoustic impedance, high compressibility, and strong nonlinear oscillations, enabling aggregation, cavitation, or rupture at low acoustic pressures [218, 219]. Under focused ultrasound (FUS), stable microbubble oscillation generates shear forces that transiently open endothelial gaps, facilitating localized drug delivery (Figure 13A(ii)) [220, 221, 222, 223, 224, 225, 226, 227].

**FIGURE 13:**
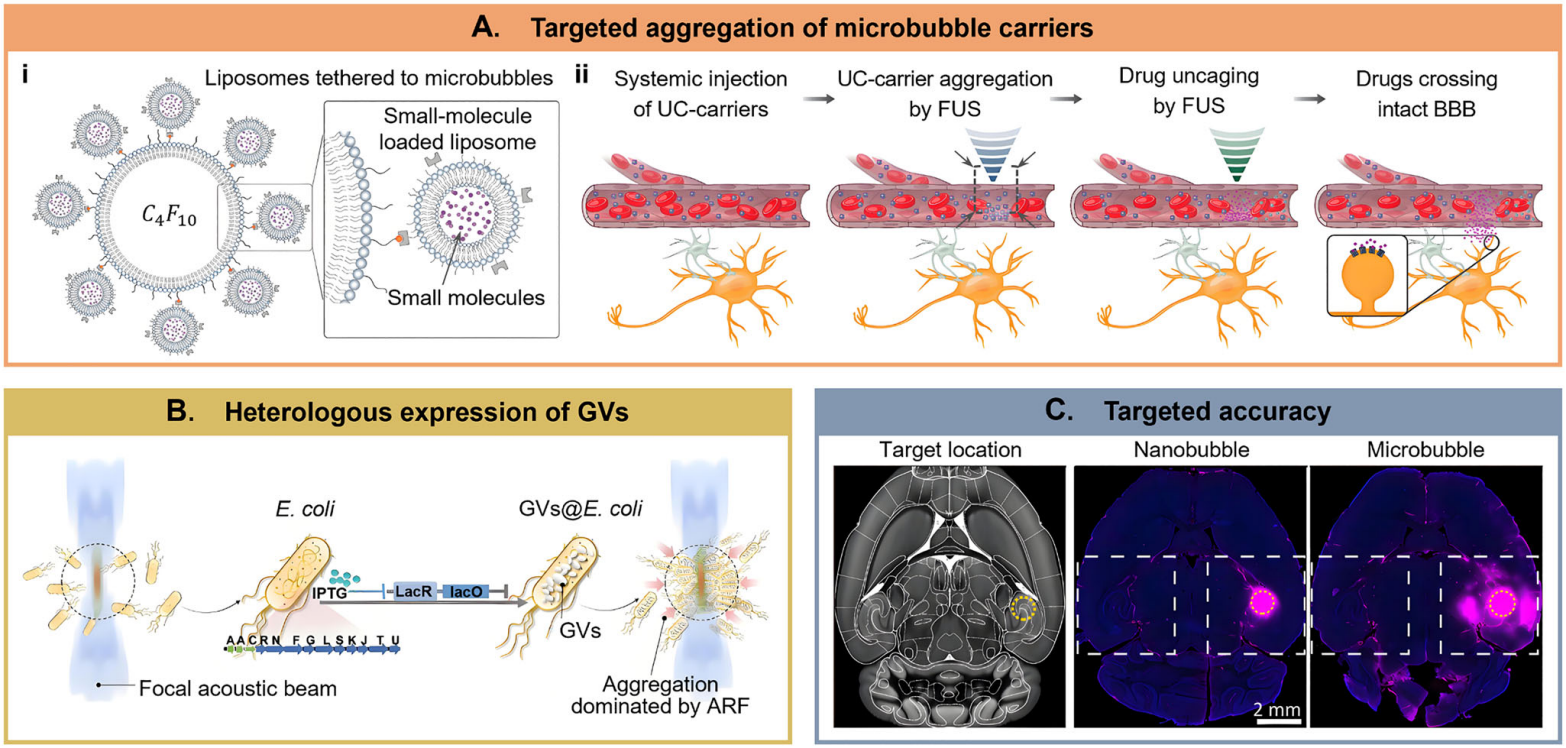
(A) (i) Small‐molecule‐loaded ultrasound‐controlled carrier (UC‐carrier) design. (ii) Concept of focal aggregation and uncaging of ultrasound‐controlled drug carriers. Reproduced with permission [217]. Copyright 2020, Springer Nature. (B) Schematic of the acoustic trapping of GVs@*E. coli*. GVs were generated in the engineered bacteria, enabling them to respond to acoustic beams and be aggregated by the acoustic radiation force. Reproduced with permission [36]. Copyright 2023, Springer Nature. (C) In vivo Evans Blue extravasation assay. The Evans Blue leakage was confined to the focused ultrasound (FUS) focal region in the nanobubble group, while additional Evans Blue leakage, indicative of side‐lobe blood–brain barrier (BBB) opening, was observed in the microbubble group. Reproduced with permission [234]. Copyright 2025, National Academy of Sciences.

Gas vesicles (GVs), which can be genetically encoded, further expand acoustic functionality. Their expression in both *Escherichia coli* (*E. coli*) and mammalian cells enables applications in imaging [228, 229, 230], biosensing [231], and targeted therapy [232]. When combined with phased‐array acoustic tweezers, GVs enable precise in vivo manipulation of engineered bacteria (Figure 13B) [36]. Compared with conventional microbubbles (∼995 nm), nanobubbles (∼273 nm) provide improved targeting precision due to their smaller size [233]. In a mouse model, they enabled targeted antibody delivery to the lateral habenula with a four‐fold increase in accuracy (Figure 13C) [234]. In addition, microbubble clusters can function as microrobots, enabling navigation in complex biological environments (Section 4.4).

Overall, bubble‐array and sharp‐edge streaming traps achieve the highest capture efficiencies for submicron bioparticles while maintaining rapid processing speeds, although they exhibit limited selectivity among similarly sized targets without biochemical functionalization. Acoustic centrifugation offers exceptional simplicity and sample economy for nanoparticle enrichment, but its throughput and droplet stability remain limiting factors. For in vivo applications, micro‐ and nanobubble‐assisted targeting enables site‐specific particle aggregation. Nevertheless, the primary challenge is balancing the acoustic pressure required for effective trapping with the biological safety window, a constraint that becomes increasingly stringent in deep‐seated or highly perfused tissues.

#### 3.3.3 Particle Sieving

Particle sieving is essential in disease diagnosis, liquid biopsy, and intercellular communication studies, requiring efficient separation across nano‐ to microscale ranges [235, 236, 237]. Acoustofluidics enables high‐performance separation of particles from complex biofluids by exploiting differences in size, density, and compressibility. Current strategies primarily use acoustic radiation forces from traveling waves [238] or pressure nodes in standing wave fields [103, 239] for high‐resolution sorting (Figure 14). For example, frequency tuning enables selective separation of polystyrene and poly(methyl methacrylate) particles [240], while focused IDTs enable continuous sorting of nanoparticles (100–500 nm) with enhanced throughput [120, 241]. Standing SAW fields achieve separation efficiencies exceeding 80% for microspheres of different sizes [53].

**FIGURE 14:**
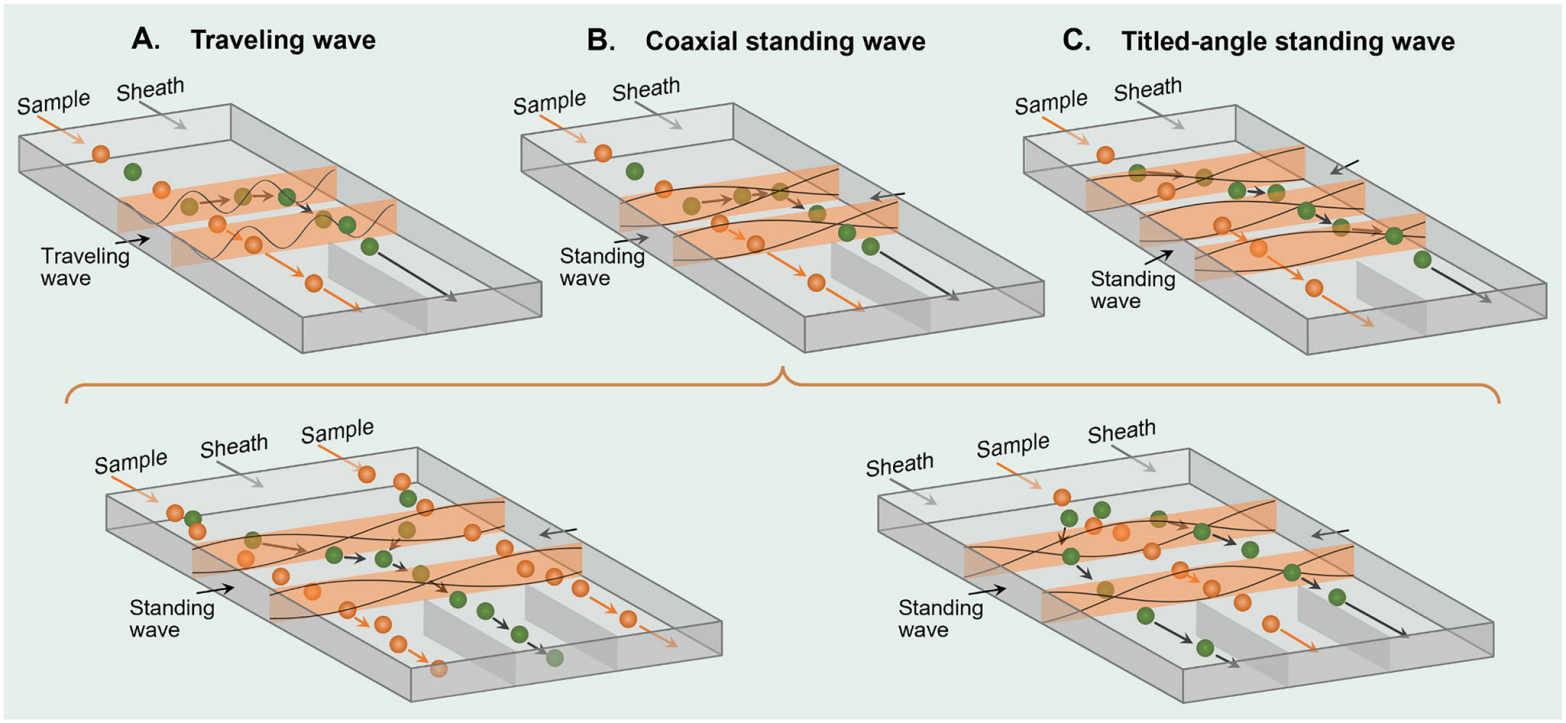
Acoustofluidic approaches for high‐throughput particle sieving. (A) Using pulsed traveling waves to separate particles. Particles can be propelled to farther distances based on differences in the acoustic radiation force acting upon them. (B) Isolating particles using a half‐wave resonator. Particles can be separated into either pressure nodes or antinodes based on differences in their physical properties. (C) Isolating particles using a tilted‐angle approach. Particles can be pushed across multiple pressure nodes, enabling larger separation distances between particles with minute differences in their physical properties.

However, conventional standing wave systems are limited to particle displacements of about one‐quarter of the wavelength. To overcome this, the tilted‐angle standing surface acoustic wave (taSSAW) approach introduces multiple pressure nodal lines, significantly enhancing lateral migration distance and separation efficiency (Figure 15A) [114, 140, 141, 142, 242]. More recently, the acoustofluidic system for targeted antibody removal in transplantation (A‐START) further demonstrates high‐throughput and selective removal of donor‐specific antibodies while preserving beneficial ones (Figure 15B) [143]. Among acoustofluidic separation strategies, taSSAW devices offer the best balance between separation resolution and throughput, achieving separation efficiencies exceeding 95% for cells and microparticles [140], with recent extensions to submicron targets such as exosomes. However, all platforms are fundamentally constrained by a trade‐off between separation resolution and throughput: improving resolution requires either longer acoustic interaction lengths or lower flow rates, whereas increasing flow rates introduces inertial effects that compromise sorting performance. In addition, complex biofluids, such as whole blood, introduce variations in viscosity and acoustic impedance that can unpredictably degrade separation efficiency.

**FIGURE 15:**
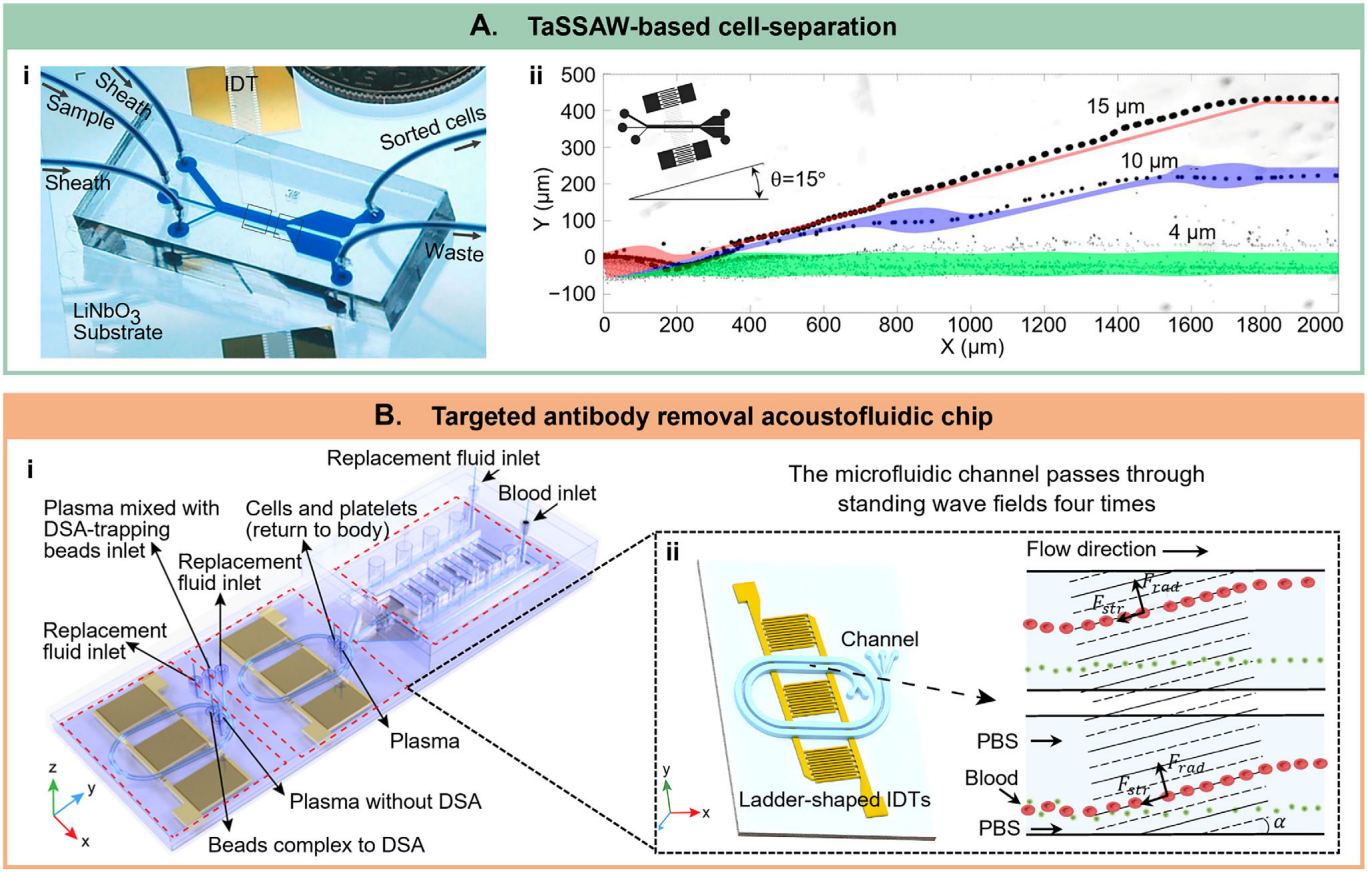
(A) (i) Photo showing a tilted‐angle standing surface acoustic wave (taSSAW)‐based cell‐separation device. (ii) Numerical simulation and experimental demonstration of particle separation processes for different sizes. Reproduced with permission [140]. Copyright 2014, National Academy of Sciences. (B) (i) The schematic of the acoustofluidic system for targeted antibody removal in transplantation (A‐START) chip. (ii) Schematic of the ladder‐shaped IDTs in the A‐START chip. After two rounds of acoustic separation, red blood cells and exosomes in the blood gradually separate. Reproduced with permission [143]. Copyright 2025, AAAS.

High‐frequency BAW devices provide additional capabilities [187, 243, 244]. The system centers on a customized fan‐shaped ultra‐high‐frequency BAW resonator operating in the thickness‐extensional mode, which generates a highly localized array of rapidly rotating, closed 3D microvortices along the device edge—termed stereo acoustic streaming (SteAS) (Figure 16A–C) [134]. These interconnected vortices collectively define a virtual channel whose geometry is governed by the electrode pattern, with its aperture dynamically tunable via channel height, resonant frequency, and input power (Figure 16D). This system demonstrates effective manipulation of ∼200 nm particles, including exosome purification. Despite these advances, challenges remain in quantitatively modeling acoustic–particle interactions and ensuring biocompatibility, sample adaptability, and clinical reliability.

**FIGURE 16:**
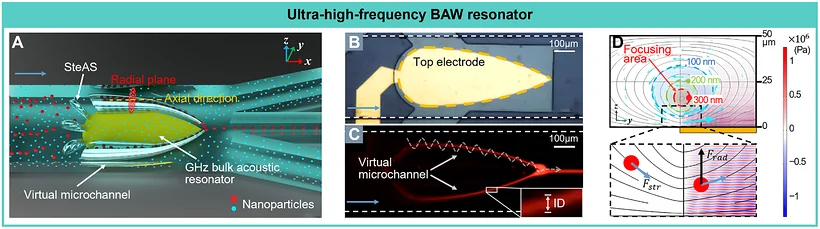
Self‐adaptive virtual microchannel for continuous enrichment and separation of nanoparticles. (A) Schematic of nanoparticle separation based on the virtual microchannel. The stereo acoustic streaming (SteAS) is triggered by a GHz BAW resonator and confined in a microchannel, forming two virtual channels composed of a series of acoustic vortices. (B) Actual image of an actual GHz BAW device integrated with a microchannel. (C) Aggregation of fluorescent PS nanoparticles in a symmetrical virtual microchannel pointed by white arrows. The inner diameter (ID) of the virtual channel is shown in the detailed image. (D) Force analysis of SteAS‐based focusing. The nanoparticles are focused into the center of the vortices due to the acoustic radiation force and the drag force. The schematic shows the focusing area for 300 nm (red), 200 nm (green), and 100 nm (blue) nanoparticles. Reproduced with permission [134]. Copyright 2022, AAAS.

#### 3.3.4 Particle Patterning

Particle patterning enables precise spatial organization of particles within engineered acoustic fields across nano‐ to millimeter scales, underpinning applications in cell assembly and tissue engineering [116, 214, 245, 246, 247, 248]. Current strategies primarily employ standing waves for periodic trapping or acoustic holography for programmable 3D field shaping (Figure 17).

**FIGURE 17:**
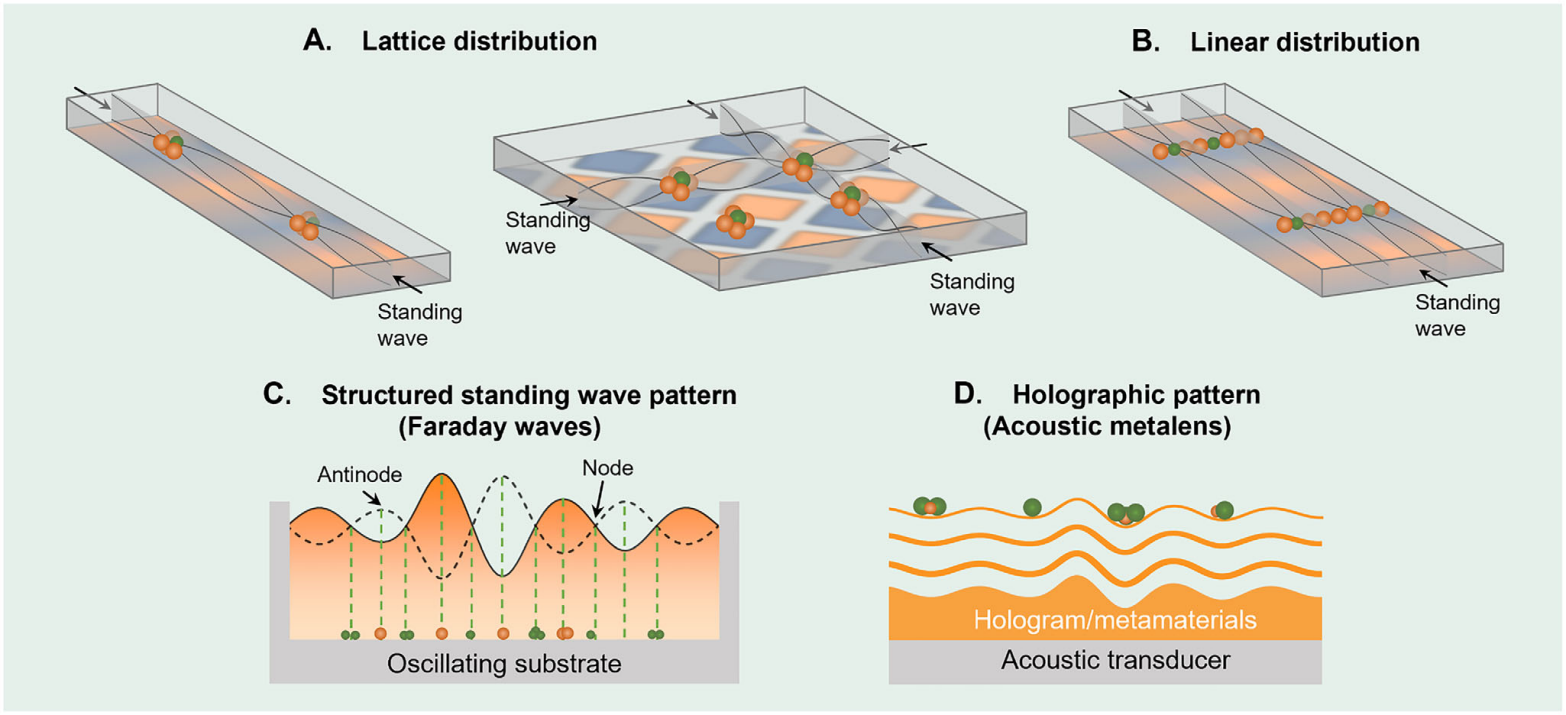
Acoustofluidic approaches for particle patterning. (A) 1D or 2D acoustic standing waves can be used to rapidly form size‐controllable particle clusters, arranged in a lattice distribution. (B) Standing waves can also be used to pattern particles and form a linear distribution. (C) Faraday wave‐based structured standing wave fields can be utilized for particle patterning. Particles can be aggregated to either pressure nodes or antinodes based on differences in their physical properties. (D) The acoustic metalens placed in front of the path acoustic transmission modulates the acoustic wave to form complex, customizable pressure distributions within the sample chamber.

*Standing‐wave BAW/SAW*: Orthogonal IDT configurations generate 2D standing SAW fields that trap particles at pressure nodes to form reconfigurable lattices (Figure 18A(i)) [249]. A key challenge is avoiding multi‐particle clusters within a single trap. Effective single‐particle isolation requires an optimal wavelength to particle diameter (λ/*d*_p_), which balances acoustic confinement and interparticle Bjerknes attraction (e.g., λ/*d*_p_≈ 3.2–3.6 for rigid particles and broader ranges for deformable cells) [59]. Building on this principle, a harmonic acoustics platform for on‐contact, dynamic, selective particle manipulation was developed to facilitate dynamic tuning of acoustic wells via segmented IDTs, allowing programmable assembly of colloidal structures and sub‐wavelength control of cell spacing (Figure 18A(ii)) [250]. Standardized protocols further support high‐throughput and reproducible single‐cell manipulation [157]. Beyond lattice assembly, acoustofluidic platforms can generate fibrous and anisotropic architectures, enabling the formation of aligned muscle tissues within collagen hydrogels under the standing wave fields [251]. An acoustofluidic bioassembly‐induced morphogenesis strategy allows cells to rapidly aggregate and organize into ordered linear or complex structures within seconds (Figure 18B(i)), thereby enhancing cell fusion, neuromuscular junction formation, contractility, and electrophysiological function, with demonstrated therapeutic efficacy in models of volumetric muscle loss [144]. Hybrid standing wave approaches that integrate surface and bulk acoustic waves further extend these capabilities to the construction of vascular networks within hydrogels (Figure 18B(ii)) [252]. Notably, high‐frequency surface acoustic waves can excite standing bulk acoustic modes in resonant cavities, overcoming intrinsic frequency limitations and enabling strong trapping with low power input [88, 99, 101, 253].

**FIGURE 18:**
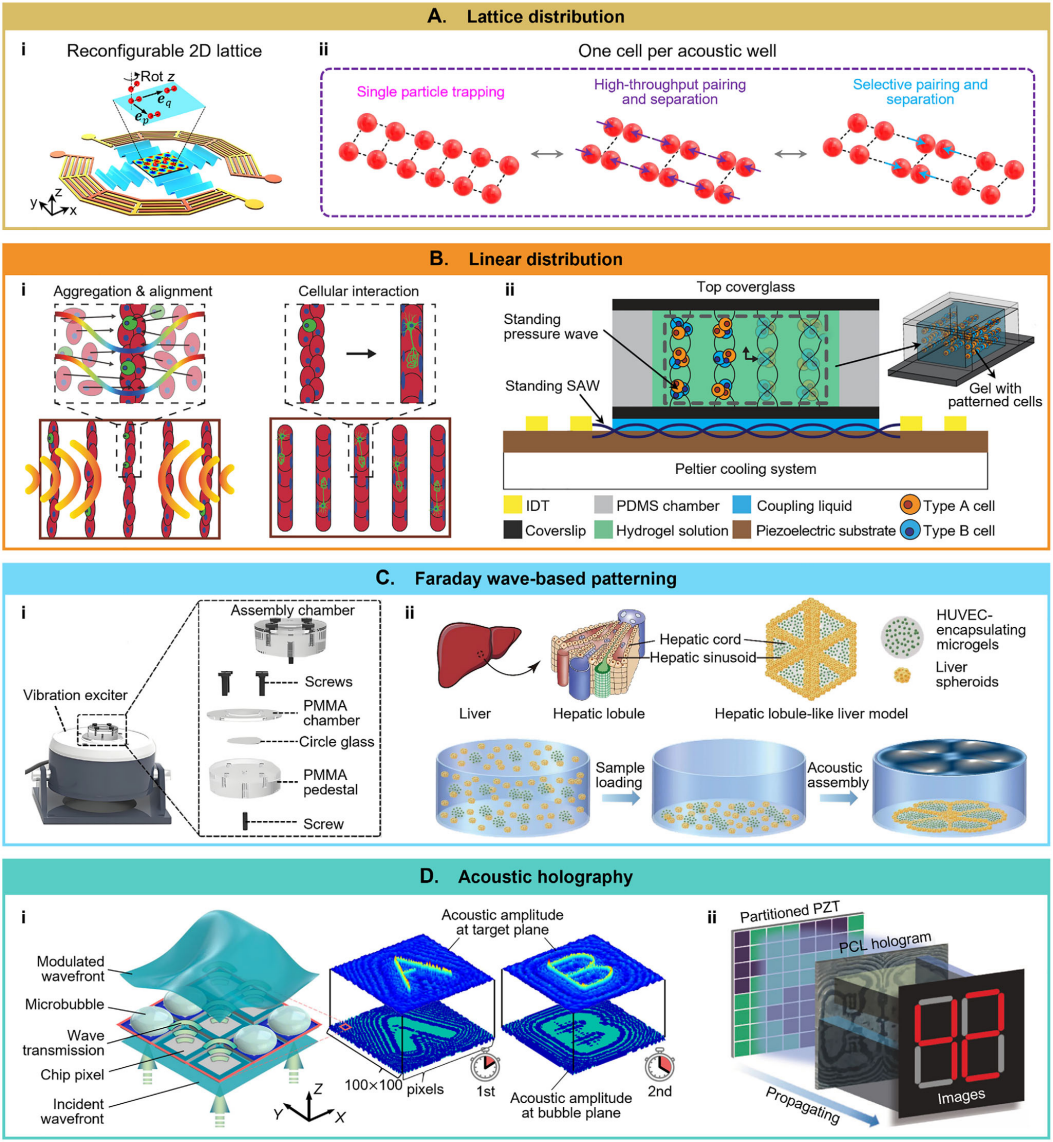
(A) (i) The designed proof‐of‐concept wave number–spiral acoustic tweezers: dynamic and reconfigurable particle manipulation. Reproduced with permission [249]. Copyright 2019, AAAS. (ii) Fourier synthesis of harmonic acoustic waves to create soft, flexible lattices for colloidal crystals or cell–cell pairing and separation. Reproduced with permission [250]. Copyright 2022, Springer Nature. (B) (i) Schematic of the cellular arrangement during acoustofluidic bioassembly‐induced morphogenesis in the neuromuscular tissues. Reproduced with permission [144]. Copyright 2025, Springer Nature. (ii) Acoustophoretic tissue fabrication system is designed to create a pattern of multi‐type cells in a 3D hydrogel matrix. Reproduced with permission [252]. Copyright 2018, Springer Nature. (C) (i) Schematic of the Faraday wave bioassembly platform. Reproduced with permission [21]. Copyright 2024, Wiley‐VCH. (ii) The cytoarchitecture of a hepatic lobule (top). Scheme of construction of a hepatic lobule model by using acoustic differential bioassembly (bottom). Reproduced with permission [257]. Copyright 2022, IOP Publishing Ltd. (D) (i) Schematic of the spatial reconfigurable ultrasound modulation based on microbubble patterns. Refreshing the microbubble pattern enables dynamic spatial ultrasound modulation. Reproduced with permission [145]. Copyright 2020, Springer Nature. (ii) Reconfigurable dynamic acoustic holography using a programmable semi‐crystalline polymer film and a partitioned PZT. Reproduced with permission [156]. Copyright 2025, Springer Nature.

*Faraday standing waves*: As a distinctive particle patterning strategy, Faraday waves provide a distinctive route for particle patterning, in which low‐frequency oscillations (40–200 Hz) generate well‐defined geometric patterns at fluid interfaces. Surface deformation‐induced circulatory flows drive suspended particles toward stable equilibrium positions, yielding ordered assemblies that can be tuned by vibration parameters, cavity geometry, and fluid properties (Figure 18C(i)) [21]. This liquid‐templated strategy enables rapid, large‐scale assembly of microscale components into symmetric and reconfigurable structures, with dynamic control achieved through modulation of frequency and acceleration [22, 254, 255]. By exploiting differences in size and buoyant density, heterogeneous cell populations can be spatially organized, enabling the construction of functional tissue models [256]; for example, acoustically assembled liver lobule structures recapitulate key hepatic functions in vitro (Figure 18C(ii)) [257]. Owing to its high efficiency and real‐time tunability, Faraday wave‐based patterning has become a versatile platform for assembling microscale materials and living systems.

*Holographic acoustic field*: Although standing wave fields can generate complex 3D patterns, their inherent periodicity limits structural diversity. Acoustic holography overcomes the intrinsic periodicity of standing waves by enabling arbitrary wavefront engineering for complex 3D patterning [37, 258, 259]. Static holographic plates encode phase information to generate prescribed acoustic fields, as demonstrated by early implementations combining iterative angular spectrum methods with 3D‐printed holograms [82]. Subsequent advances have enabled true 3D matter assembly through superposition of holographic fields, with applications in microfabrication and tissue engineering [255]. Recent efforts focus on enhancing resolution and reconfigurability via intelligent algorithms and advanced materials, including metamaterials and metasurfaces [117, 260, 261, 262]. For instance, programmable microbubble arrays dynamically modulate acoustic fields through impedance contrast (Figure 18D(i)) [145], while reconfigurable platforms based on phase‐change materials, such as crosslinked poly(ε‐caprolactone) films integrated with partitioned transducers, enable real‐time wavefront control at ultrafast speeds (up to 50 000 fps) (Figure 18D(ii)) [156]. Despite remaining challenges in resolution, material compatibility, and full‐field programmability, continued advances in materials design and artificial intelligence (AI)‐assisted optimization are expected to transform acoustic holography into an adaptive and intelligent platform for acoustic field control.

Particle patterning relies on three primary acoustic strategies: periodic lattice assembly using standing‐wave fields (BAW or SAW), interfacial patterning via Faraday waves, and arbitrary 3D structure formation through acoustic holography. Standing‐wave methods are the most mature for high‐throughput parallel assembly but are inherently limited by field periodicity and restricted pattern flexibility. Faraday‐waves approaches enable rapid assembly of cells and soft materials at liquid‐air interfaces, although pattern controllability and spatial resolution remain constrained by liquid‐layer thickness and vibration parameters. Acoustic holography offers the greatest design freedom and 3D complexity is limited by slow dynamic reconfiguration and high system complexity. Across all approaches, maintaining pattern fidelity at high throughput remains a major challenge. From a mechanistic perspective, quantitative predictive models are still needed to describe the competition between acoustic radiation forces and secondary Bjerknes forces in dense particle assemblies, as well as the influence of hydrodynamic interactions on final particle organization.

## 4 Acoustic Propulsion of Microswimmers

Acoustically driven microswimmers are particles capable of autonomous locomotion under acoustic actuation. Here, autonomous refers to the self‐sustaining conversion of locally available energy (e.g., from a global acoustic field, chemical gradients, or ambient sources) into kinetic motion without tethered power, rather than to onboard decision‐making or artificial intelligence. Owing to their small size, wireless controllability, and efficient propulsion in low‐Reynolds‐number regimes, these systems hold considerable promise for biomedical applications, including targeted drug delivery [263, 264], diagnosis [265, 266], and biosensing. A central design constraint arises from the low‐Reynolds‐number regime, where viscous forces dominate over inertia. At microscale dimensions, *Re*_str_ is typically very small and governed primarily by swimmer size, rendering inertial effects negligible relative to viscous drag and Brownian motion. Under these conditions, time‐reversible (reciprocal) motions cannot produce net propulsion—a constraint formalized by the scallop theorem (Figure 19A) [267]. Effective locomotion therefore requires non‐reciprocal or symmetry‐breaking actuation strategies. Current acoustically driven microswimmers achieve propulsion through mechanisms such as geometric or density asymmetry, acoustic cavitation, and sharp‐edge oscillations, which generate asymmetric acoustic radiation forces or localized streaming flows (Figure 19B).

**FIGURE 19:**
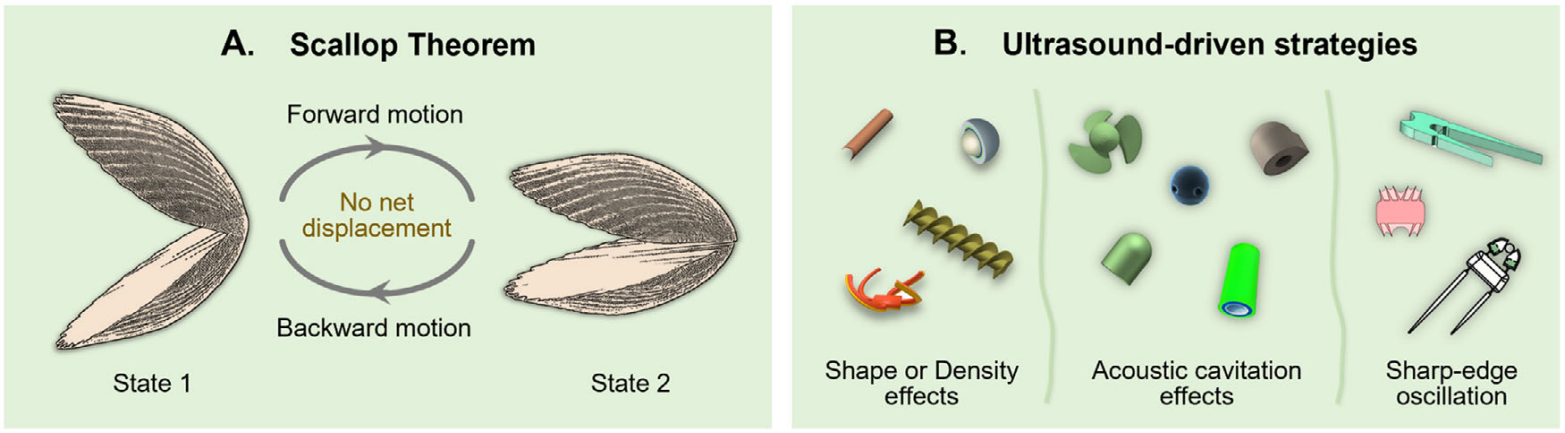
(A) Schematic drawing of the “Scallop Theorem.” A motor relying on such a simple back‐and‐forth symmetric motion cannot overcome the viscous drag forces and hence will not generate net directional displacement at the microscale. (B) Schematic illustrating the ultrasonic driving strategy for microswimmers.

### 4.1 Shape or Density Effect

Geometric and density asymmetries provide a primary route for acoustic propulsion at low Reynolds numbers. Table 5 summarizes representative examples within this design space, highlighting their size regimes, acoustic operating parameters, and key functional capabilities. Early studies of asymmetric nanorods with concave–convex ends attributed directed motion to “self‐acoustophoresis” [268, 269], in which curvature‐induced scattering concentrates acoustic energy and generates a propulsion‐driving pressure gradient (Figure 20A). However, subsequent observations of reversed motion challenged this model [270], leading to the development of an “asymmetric steady streaming” framework. In this view, oscillatory particle motion in a viscous fluid generates inertia‐induced steady streaming, producing asymmetric surface stresses and net thrust perpendicular to the oscillation direction [271]. A generalized theoretical framework has since shown that both shape and density asymmetries jointly govern propulsion direction and stability [272], with enhanced directional persistence at high acoustic Reynolds numbers or strong asymmetry. Experimental studies, including Janus particles, confirm that density asymmetry alone can drive propulsion, with speed determined by asymmetry, acoustic frequency and amplitude, and fluid viscosity [273, 274]. Together, these findings establish a robust design basis for controllable and efficient acoustically driven micro‐ and nanomotors.

**TABLE 5:** Summary of representative acoustically driven microswimmers based on shape or density asymmetry.

Shape/Design	Size	Preparation	Acoustic conditions	Functions/Tasks	Refs.
Metallic microrods	2 µm in length, 330 nm in diameter	Template electrodeposition	Standing BAWs: 3.7 MHz frequency, 10 V_pp_ voltage	Axial directional motion (up to ∼200 µm s^−1^), in‐plane rotation, chain assembly, axial spinning, and pattern formation	[268]
Metallic nanowires	4 µm in length, 200 nm in diameter	Template electrodeposition	Standing BAWs: 2.66 MHz frequency, 6 V_pp_ voltage	Extracellular autonomous propulsion, penetration of cell membranes, intracellular rotational movement; Intracellular siRNA delivery	[282]
Twisted star shape	5.8–23 µm in diameter, 150 nm in thickness	Projection lithography, thermal evaporation	Standing BAWs: 3.77 MHz frequency, 10 V_pp_ voltage	Directional rotation (determined by chirality and the number of arms)	[275]
3D twisted nanocrystal bimorphs	square body (1.5 µm wide), 3D curvature of the arms (2.5 µm arc length, 60 nm wide), arm heights (0/0.5/1.35 µm)	Nanoimprint lithography, nanocrystal assembly	Standing BAWs: 2.2/3.4/5.3 MHz frequency	Rotate around an axis perpendicular to the substrate; 3D complex phase separation	[276]
Cylindrical core & double‐helical vane	350 µm in length, 100 µm in diameter	Two‐photon lithography	Traveling BAWs: 11–19 kHz frequency, 15–60 V_pp_ voltage	Spiral propulsion (rotate around a long axis while simultaneously translating along the axis); Bi‐directional controllable motion, navigation in 3D architecture	[281]

**FIGURE 20:**
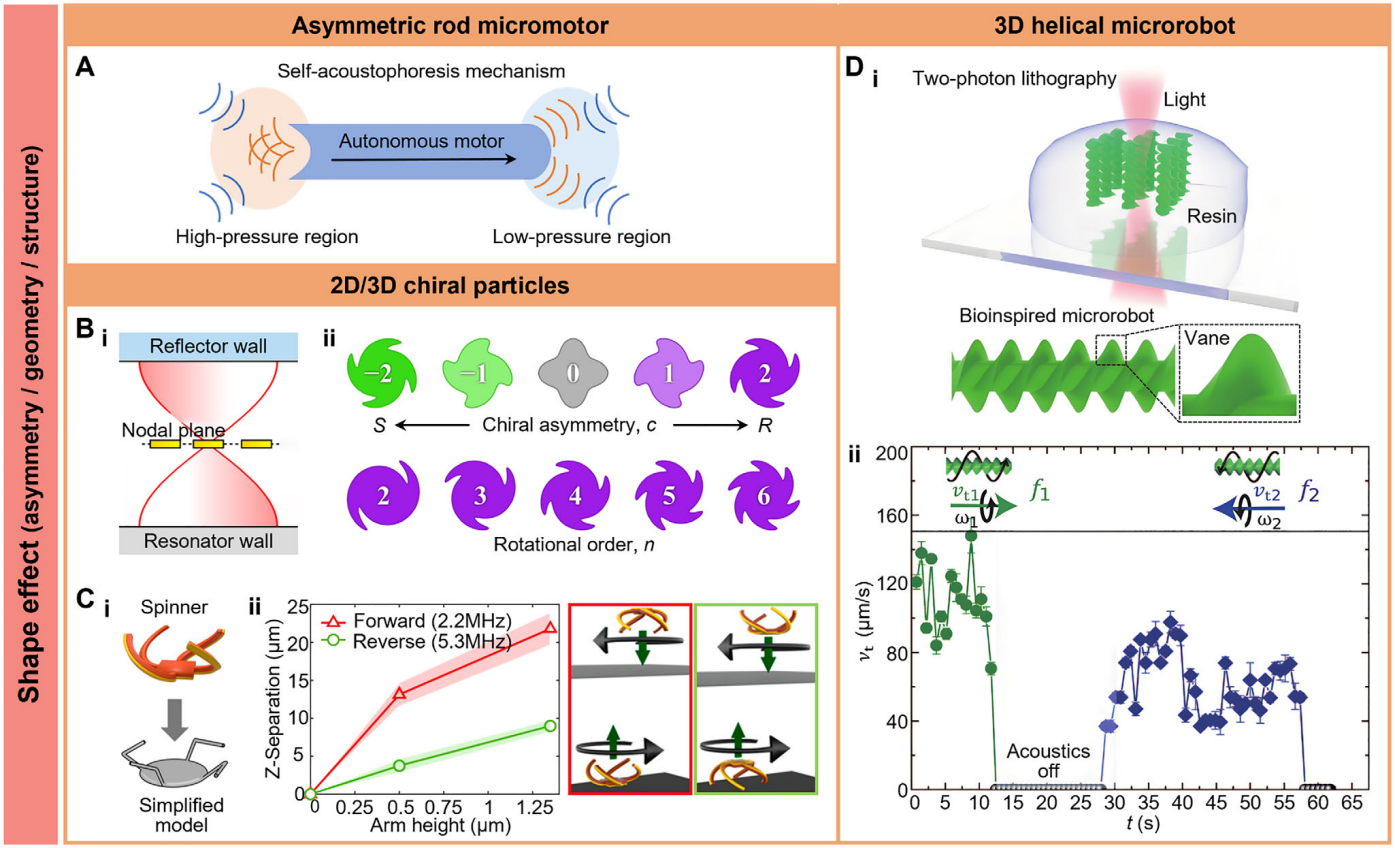
(A) Self‐acoustophoresis mechanism: curvature‐induced acoustic pressure gradient. (B) (i) The standing wave acoustic pressure gradient caused the rapid migration of gold particles to the nodal plane. (ii) Particle shapes for different values of the chiral asymmetry *c* and the rotational order *n*. Reproduced with permission [275]. Copyright 2018, American Chemical Society. (C) (i) Real rotator geometry and its simplified model. (ii) Height separation between oppositely oriented spinners as a function of the arm height for forward (red) and reverse (green) spinners. Reproduced with permission [276]. Copyright 2023, American Chemical Society. (D) (i) The acoustic helical microrobots are mass‐manufactured using the two‐photon lithography technique. The bottom inset shows an illustration of a bioinspired robot. (ii) The plot illustrates the speed profile of the microrobot during its bidirectional trajectory. Reproduced with permission [281]. Copyright 2023, AAAS.

Chirality introduces an additional degree of control, enabling complex rotational and collective dynamics. Chiral microstructures, such as twisted gold microplates, exhibit rotation behaviors that depend on both handedness and geometric features, e.g., fin number (Figure 20B) [275]. More broadly, acoustic fields can induce dynamic self‐organization and multidimensional phase separation of chiral spinners, extending active matter studies into three dimensions (Figure 20C) and enabling chirality‐based assembly and separation strategies [276]. For microswimmers driven by traveling acoustic waves, propulsion performance depends sensitively on geometric parameters, including shape, aspect ratio, and orientation [277, 278, 279, 280]. However, purely acoustic control of three‐dimensional navigation remains challenging, and most current approaches rely on auxiliary magnetic fields for orientation. Notably, acoustically driven helical microrobots consisting of a cylindrical core and a double‐helical vane have demonstrated frequency‐controlled bidirectional motion without reorientation (Figure 20D) [281], highlighting the potential of coupling structural symmetry with acoustic microstreaming for robust navigation in complex environments.

The mechanisms discussed above operate primarily in the micrometric regime (*a* ≈ 1–10^2^ µm), where *a*  ≫  δ. In this condition, the viscous boundary layer is negligibly relative to the swimmer size, allowing inertia‐driven steady streaming or asymmetric scattering to generate efficient thrust. As the swimmer shrinks to the submicron regime, however, the propulsion mechanism changes fundamentally: viscous damping severely suppresses inertia‐driven streaming, while Brownian motion becomes non‐negligible. Consequently, shape‐ or density‐induced asymmetries alone are insufficient to produce effective thrust, resulting in a dramatic decline in propulsion efficiency.

### 4.2 Acoustic Cavitation Effect

Acoustic cavitation provides a powerful propulsion mechanism by exploiting the strong, localized flow fields generated by oscillating bubbles [283]. Bubble‐based microswimmers typically incorporate microcavities that trap gas pockets, which undergo volumetric oscillations under acoustic excitation. When the oscillatory flow attains sufficiently high velocity, asymmetric fluid exchange at the cavity opening produces net momentum flux and a recoil force that drives propulsion [284, 285]. The localized microstreaming intensity around oscillating bubbles can be characterized by the interfacial Reynolds number

(11) $$Re_{\mathrm{int}}=4\pi rf\varepsilon /\mu$$

where *r* is the bubble radius, *f* the excitation frequency, and ε the radial displacement amplitude. The interfacial Reynolds number typically exceeds that of whole‐body swimming by several orders of magnitude, enabling strong propulsion. The resulting thrust scales with microstreaming velocity and can be tuned through bubble resonance, cavity geometry, and fluid properties.

Resonance behavior plays a central role in propulsion efficiency. Predictive models describe the dependence of resonance frequency on geometric and material parameters, including surface tension effects that become dominant at micrometer scales [286, 287]. At resonance, propulsion scales quadratically with oscillation amplitude and thus with input voltage, providing an effective means of control [288]. However, as swimmer size decreases, reduced Reynolds number limits thrust, necessitating high oscillation velocities to maintain performance [285]. Table 6 summarizes representative cavity‐based microswimmers, including their fabrication methods, acoustic operating parameters, and demonstrated functionalities.

**TABLE 6:** Summary of representative acoustically driven microswimmers based on acoustic cavitation.

Shape/Design	Size	Preparation	Acoustic conditions	Functions/Tasks	Refs.
Microtubule complex	80–100 µm in diameter, 470/890/590 µm in length	Two‐photon polymerization 3D printing	Traveling BAWs: 11.7/5.9/7.9 kHz frequency	3D spatial navigation, autonomous attitude recovery, frequency‐selective control	[291]
Tubular (double‐opening conical cavity)	5 µm in diameter, 10 µm in length, 3.5–4 µm in inner opening	Template‐assisted electrochemical deposition	Traveling BAWs: 4.6 MHz (bubble resonance), 21 kHz (aggregation) frequency, 15 V_pp_ voltage	Motion modes: linear, spiral, circular, etc.; controlled aggregation/dispersion Enhancement of local fluorescence signal (biological detection)	[302]
Rectangular body	∼100 µm in pit diameter, 150–250 µm in pit length	Ultraviolet photopolymerization	Traveling BAWs: 20–22 kHz frequency, 5–15 V_pp_ voltage	Linear/rotational motion (magnetically controlled)	[292]
Propeller‐shaped (spherical cavity, asymmetrically distributed arc fins)	Spherical cavity: 11 µm in diameter, 7.4 µm in orifice diameter	Two‐photon polymerization 3D printing	Traveling BAWs: 320 kHz frequency	Rotation, orbital movement (up to ∼2855 µm s^−1^ (about 143 body length s^−1^)); Physical anchoring, drug sustained release	[301]
Spherical shell (eccentric cavity with two orifices (included angle of 90°))	30 µm in outer diameter, 18 µm in cavity diameter	Two‐photon polymerization 3D printing	Focused ultrasound: 4480 kHz frequency	Magnetically guided motion; Real‐time ultrasonic imaging, drug targeted delivery	[303]
Spherical shell	72.6 µm in outer diameter, 3.5 µm in the wall thickness, 25 µm in orifice diameter	Two‐photon polymerization 3D printing	Traveling BAWs: 95–108 kHz frequency, 7–14 Vpp voltage	Oriented movement along the cavity wall; Drug delivery, real‐time ultrasound imaging (enhanced imaging signal)	[304]
Triangular (with two bubble cavities on the back)	18 µm in cavity diameter, 9/10 µm in orifice diameter	Two‐photon polymerization 3D printing	Traveling BAWs: 300–345 kHz (linear translation), 215–235 kHz (rotation) frequency	Linear translation, rotation, surface‐slipping	[305]
Symmetrical array of inclined cylindrical bubble cavities	Bubble cavity: 10 µm in cavity diameter, 100 µm in length	Two‐photon polymerization 3D printing	Traveling BAWs: 41 kHz frequency	Magnetically guided motion	[306]
Half‐capsule shape	5 µm in outside diameter, 7.5 µm in length, 500 nm in the wall thickness	3D direct laser lithography, vertical electron beam deposition	Traveling BAWs: ∼1.33 MHz frequency, 4 kPa acoustic pressure	Translate along the boundary (magnetic orientation); Non‐contact manipulation of individual particles and cells	[286]
Cup‐shaped	Template spheres: 3 µm, 1 µm, 600 nm, 500 nm	Nanosphere lithography, reactive ion etching (RIE)	Traveling BAWs: ∼1.45 MHz (3 µm), ∼18 MHz (500 nm) frequency	Magnetically guided 2D motion, autonomous 3D motion; Non‐contact manipulation of individual particles and cells	[298]
Bullet‐shaped (the side wall is provided with a fin)	24 µm in diameter, 9 µm in bubble diameter, 3.5 µm in orifice diameter, 25 µm in length	Two‐photon polymerization 3D printing	Traveling BAWs: 327 kHz frequency, 3.5–8 Vpp voltage	Slip along the wall (magnetic orientation); Particle transport	[299]
Bullet‐shaped (the side wall is provided with a fin; double reentrant orifice structure)	30 µm in diameter, 18 µm in bubble diameter, 6 µm in orifice diameter, 27 µm in length	Two‐photon polymerization 3D printing	Traveling BAWs: 380 kHz frequency, 1–4 Vpp voltage	Surface‐slipping (Newtonian fluid), puller‐type propulsion (non‐Newtonian fluid)	[300]

Exploiting the size‐dependent resonance of bubbles enables frequency‐selective activation of tubular cavities, allowing directional navigation of microswimmers in three‐dimensional microfluidic environments [289, 290, 291]. However, as dimensions decrease, control based solely on resonance differentiation becomes increasingly limited. To overcome this constraint, magnetic functionality has been introduced by embedding superparamagnetic particles within polymer matrices [292] or depositing thin Ni layers, enabling external field‐guided steering. For example, half‐capsule microswimmers fabricated via 3D direct laser lithography with subsequent Ni/Au deposition exhibit bubble resonance under acoustic excitation (1.1–1.4 MHz) [286]. Near rigid boundaries, acoustic scattering induces secondary Bjerknes forces that promote strong wall adhesion [293, 294, 295], suppressing propulsion; this effect can be mitigated by applying a magnetic field to tilt the swimmer and restore net thrust through force rebalancing (Figure 21A).

**FIGURE 21:**
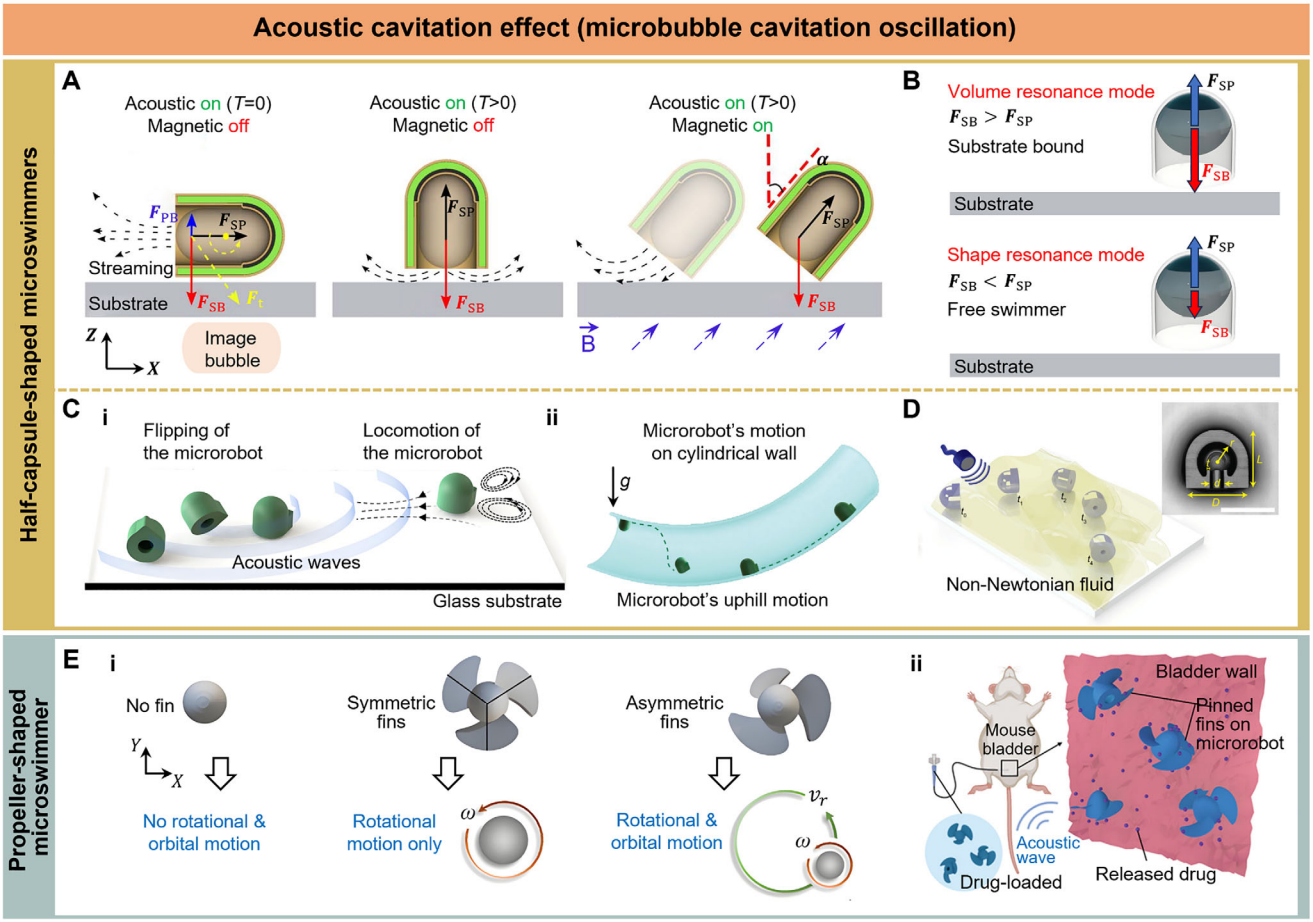
(A) Schematic illustration of the forces acting on an acoustically driven microswimmer upon application of an acoustic field (*T*  =  0), including the primary Bjerknes force *F*_PB_ (blue solid arrow), secondary Bjerknes force *F*_SB_ (red solid arrow), and streaming propulsive force *F*_SP_ (black solid arrow). The resultant force (yellow dashed arrow) generates a torque that rotates the swimmer axis toward the *z* direction. While wall‐adsorbed, the microswimmer remains stationary. Application of an external magnetic field (blue dashed arrows) reorients the swimmer, restoring a net propulsion force and initiating translation. Reproduced with permission [286]. Copyright 2019, AAAS. (B) Force balances and locomotion modes across different bubble vibration regimes. Under shape‐resonance conditions, microswimmers generate sufficient thrust to overcome wall adhesion, enabling 3D free‐swimming locomotion. Reproduced with permission [298]. Copyright 2020, American Chemical Society. (C) (i) Under the acoustic waves, the robot flips toward the substrate and slips forward. Asymmetric fins break the symmetry of the surrounding flow field. (ii) Schematic of the surface slipping of the microbubble‐based microrobot on curved boundaries inside circular channels. Reproduced with permission [299]. Copyright 2020, National Academy of Sciences. (D) Puller‐type acoustic propulsion of the microrobot in non‐Newtonian fluids. The inset shows a confocal image of the 3D cross‐sectional profile of the microrobot. Reproduced with permission [300]. Copyright 2022, AAAS. (E) (i) Schematic illustration of microrobot designs and corresponding motions under acoustic excitation. Asymmetric fins are used to break the symmetry of axial rotation. (ii) Illustration of drug‐loaded microrobots tempering an inflammatory response in bladder tissue. Fins improve their pinning against the wall. Reproduced with permission [301]. Copyright 2023, Wiley‐VCH.

Structural design further expands operational versatility [296, 297]. Half‐capsule swimmers (∼500 nm) can be produced via wafer‐scale, lithography‐free approaches, while exploiting shape resonance [190]—rather than volumetric resonance—enables low‐frequency excitation, sufficient propulsion, and reduced wall confinement, thereby favoring autonomous three‐dimensional motion (Figure 21B) [298]. Conversely, capsule‐shaped swimmers can harness wall adhesion to achieve vertical self‐alignment and rapid surface‐guided locomotion (Figure 21C) [299]. The incorporation of asymmetric fin structures breaks the balance between acoustic streaming propulsion and adhesion, enabling magnetically guided directional control. Additional refinements, such as double re‐entrant cavity geometries, enhance bubble stability and extend operational lifetimes without chemical stabilization [300]. These swimmers generate localized, high‐shear cavitation microflows that induce anisotropic deformation in viscoelastic media, supporting efficient propulsion in complex biological fluids, including blood and mucus (Figure 21D). In vivo demonstrations further highlight their potential: finned bubble‐powered robots achieve stable tissue adhesion and controlled drug release in mouse bladder models (Figure 21E) [301].

Cavitation‐driven propulsion is the most powerful and versatile mechanism currently available for acoustic microswimmers. Localized microstreaming generated by resonantly oscillating bubbles readily overcomes viscous drag in high‐viscosity biofluids, while resonance tuning enables frequency‐selective actuation of individual swimmers within multi‐robot systems. Nevertheless, important challenges remain. Propulsion efficiency decreases rapidly with swimmer size, and bubble stability and lifetime under physiological conditions remain difficult to control. A key advantage of bubble‐based microswimmers is their intrinsic ultrasound contrast, which enables real‐time imaging and feedback control. This capability distinguishes them from other acoustic propulsion strategies and makes them particularly promising for clinical translation.

Real‐time visualization is essential for translating microswimmers into minimally invasive biomedical applications, particularly within fluid‐filled cavities such as the gastrointestinal tract, bladder, vasculature, and central nervous system. Owing to the pronounced acoustic impedance mismatch between gas and surrounding tissues, bubbles act as intrinsic ultrasound contrast agents, conferring a distinct advantage to bubble‐powered microswimmers that integrate propulsion and imaging [233, 307, 308]. For instance, biodegradable hydrogel‐based microrobots with eccentric dual‐orifice cavities can be tracked in vivo using ultrasound imaging, enabling real‐time monitoring of spatial distribution (Figure 22A) [303]. Complementary approaches, such as color flow mapping (CFM) based on pseudo‐Doppler shifts [309] generated by high‐frequency oscillations at the bubble–liquid interface, further enable dynamic visualization of microswimmer motion (Figure 22B) [304]. Continued advances in fabrication, propulsion control, imaging integration, biocompatibility, and therapeutic performance are accelerating the transition of acoustically driven microswimmers from proof‐of‐concept systems toward clinically relevant platforms for targeted drug delivery and minimally invasive intervention [310, 311, 312, 313].

**FIGURE 22:**
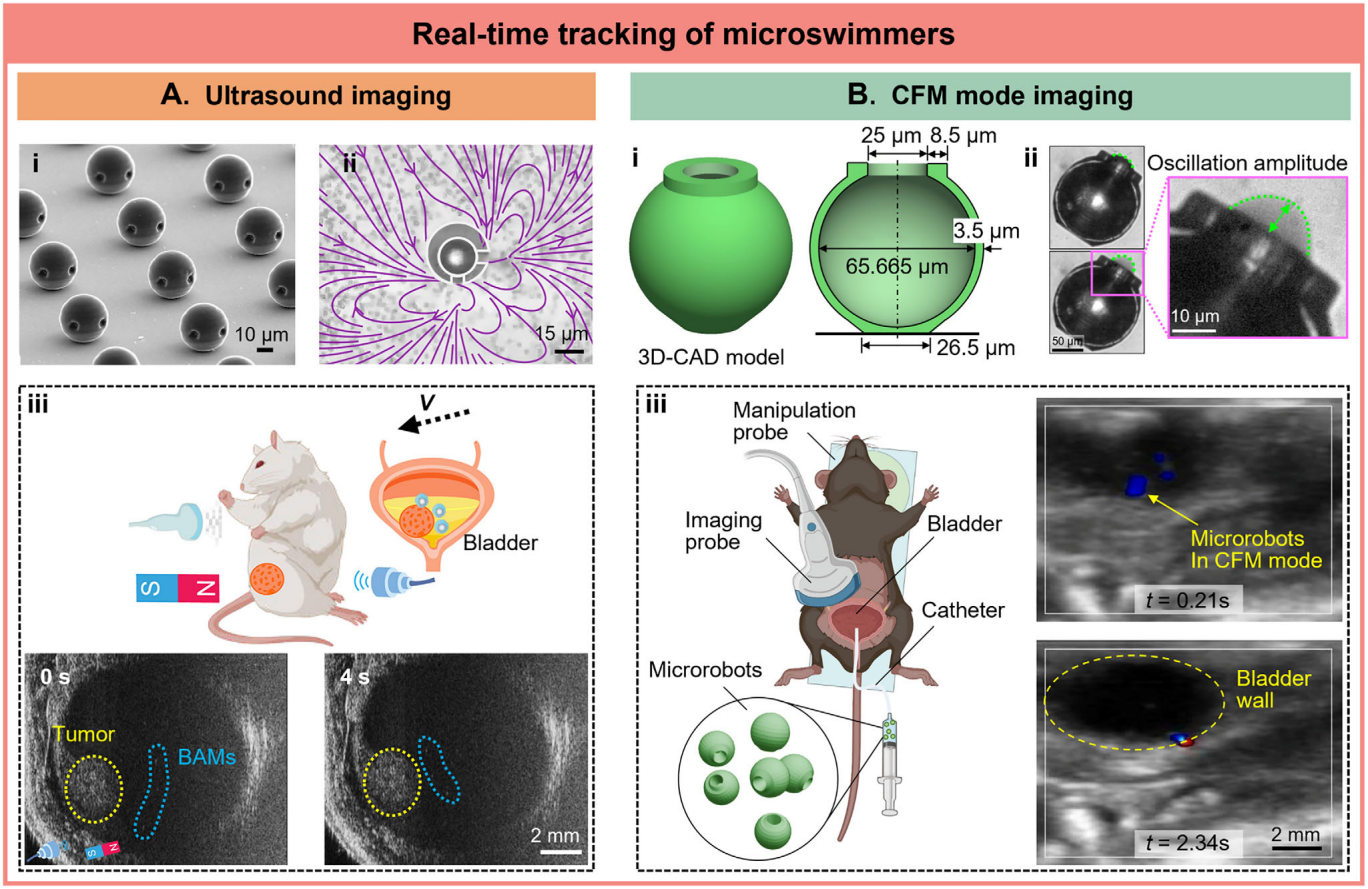
(A) (i) SEM characterization of 3D‐printed dual‐opening bioresorbable acoustic microrobots. (ii) The time‐averaged streamline patterns of the vortex profiles of a dual‐opening microrobot at the resonant frequency. (iii) Schematic and the corresponding ultrasound images of acoustic propulsion‐based, magnetically navigated tumor targeting of microrobots in a mouse bladder with an orthotopic bladder tumor. Reproduced with permission [303]. Copyright 2024, AAAS. (B) (i) 3D‐CAD model, cross‐sectional view, and dimensions of the bubble‐based acoustic microrobot. (ii) An entrapped microbubble is stimulated to oscillate at large amplitudes when actuated with an externally applied acoustic field. (iii) Schematic illustration of ex vivo experiment setup. The mouse model was bedded on an inverted manipulation probe. After the injection of microrobots, distinct color flow mapping signals were captured from within the dark‐colored bladder, visualizing the microrobots’ presence and motion toward the bottom bladder wall when manipulated by acoustic stimulation. Reproduced with permission [304]. Copyright 2025, AAAS.

### 4.3 Sharp‐Edge Oscillation

In nature, flagellated microorganisms such as *E. coli* and human sperm achieve propulsion through planar beating or three‐dimensional helical undulations, thereby overcoming the constraints of low‐Reynolds‐number locomotion [314, 315, 316, 317, 318]. Inspired by these strategies, acoustically driven artificial flagellated swimmers and sharp‐edge‐based devices have been developed [319, 320, 321, 322, 323]. Table 7 summarizes representative examples of artificial flagella, ciliary belts, and clamp‐shaped multifunctional architectures, highlighting their actuation frequencies, propulsion modes, and functional capabilities. For example, microswimmers equipped with flexible flagella can achieve efficient propulsion in traveling acoustic fields via small‐amplitude, high‐frequency tail oscillations (Figure 23A) [324]. Micro‐structured devices fabricated by two‐photon polymerization, including microjet engines and propellers, exhibit distinct resonance frequencies, enabling frequency‐selective activation for precise control of fluid transport, mixing, and microscale manipulation [325]. Drawing inspiration from starfish larvae, which regulate flow by reorienting ciliary bands, ultrasound‐actuated artificial cilia with anisotropic geometries have been developed to direct fluid either toward or away from the device (Figure 23B) [326]. More broadly, the resonance diversity of sharp‐edge structures enables selective actuation; for instance, dual‐tailed swimmers with unequal tail lengths can be independently activated by tuning the excitation frequency [327].

**TABLE 7:** Summary of representative acoustically driven microswimmers based on sharp‐edge oscillation.

Shape/Design	Size	Preparation	Acoustic conditions	Functions/Tasks	Refs.
Bimetallic head and a flexible tail flagellum	Flagella: 0.3–0.6 µm in diameter, 15–20 µm in length	Multistep electrodeposition techniques	Traveling BAWs: 91.5 kHz frequency	Linear motion	[324]
Head and a flexible tail flagellum	∼180 µm in length, ∼60 µm in width, ∼45 µm in height	In situ photopolymerization	Traveling BAWs: 4.6 kHz frequency, 140 V_pp_ voltage	Linear motion (up to ∼1200 µm s^−1^)	[319]
Ciliary band (starfish larva‐inspired)	Ciliary: 100 µm in length, 50 µm in height; 280µm in total width	Ultraviolet polymerization projection lithography	Traveling BAWs: ∼68.5 kHz frequency, 1–25 V_pp_ voltage	Translate (along the short axis, up to 2.6 mm s^−1^ (about 10 body length s^−1^)); Particle capture and transport	[326]
Double‐tailed structure (inspired by Chlamydomonas)	∼220 µm in length, ∼60 µm in height, ∼160µm in width; 5° in tip angle, ∼100 µm in tail spacing	Ultraviolet polymerization projection lithography	Traveling BAWs: ∼4 kHz frequency, 36–180 V_pp_ voltage	Linear motion (up to ∼200 µm s^−1^)	[320]
Swallowtail‐shaped (with two asymmetrical tails)	200/300 µm in tail length	Ultraviolet polymerization	Traveling BAWs: 9.82 kHz (linear motion), 8.89 kHz (turn left), 11.05 kHz (turn right) frequency, 100–180 V_pp_ voltage	Linear motion (up to 535 µm s^−1^), turn left/right (frequency‐regulated)	[327]
Clamp‐shaped (with two asymmetrical tails and two clamp claws)	∼883 µm in total length	Two‐photon polymerization 3D printing	Traveling BAWs: 46.4 kHz (bubble oscillation), 0.6 kHz (linear motion), 1.5 kHz (turn left), 6.7 kHz (turn right) frequency	Linear motion (up to 106 µm s^−1^), turn left/right (frequency‐regulated); Single‐particle grasping and transportation	[329]

**FIGURE 23:**
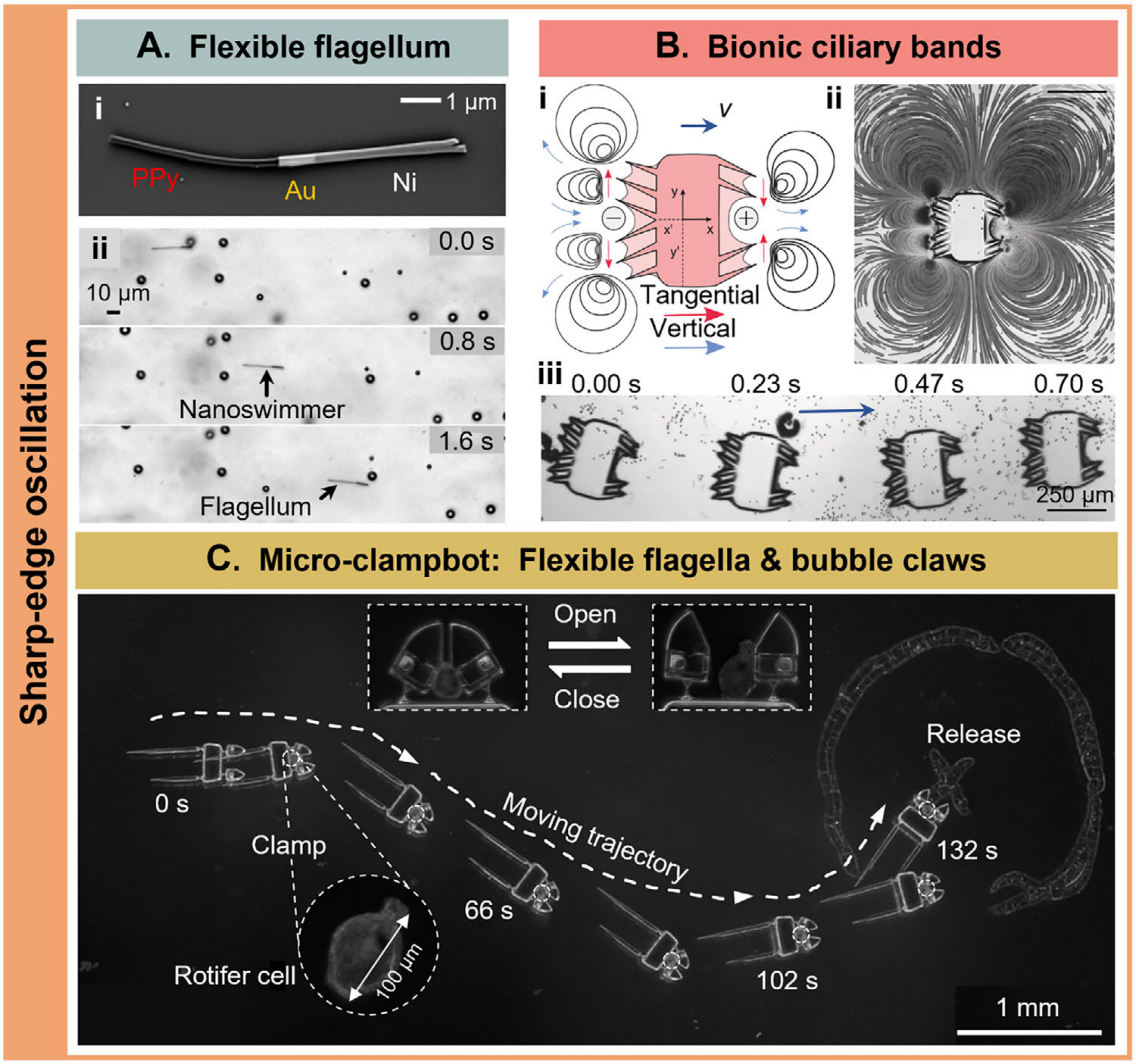
(A) (i) SEM image of a nanoswimmer. (ii) Image sequence demonstrates the translation motion of the acoustic nanoswimmer. Reproduced with permission [324]. Copyright 2016, American Chemical Society. (B) (i) Schematic of an artificial microrobot consisting of a “–” ciliary band on the left and a “+” ciliary band on the right. (ii) Image sequence illustrating the streaming flow profile. (iii) Superimposed time‐lapse image of controlled translation motion of a microrobot. Reproduced with permission [326]. Copyright 2021, Springer Nature. (C) The micro‐clampbot successfully captured a rotifer cell and transported it to the target position. Reproduced with permission [329]. Copyright 2025, AAAS.

To address the demand for multifunctional microrobots in biomedical applications, integrated systems capable of grasping, transporting, and releasing micro‐objects have recently emerged. A representative example is the acoustically driven micro‐clampbot, comprising paired claws [205, 328], dual flagella, and a compliant central body (Figure 23C) [329]. Differential frequency actuation of the flagella enables programmable locomotion modes, including adaptive navigation and real‐time obstacle avoidance. Experimentally, the device traversed channels as narrow as 2.1 body widths and transports delicate biological cargo, such as 100 µm rotifers, at speeds up to 106 µm s^−1^ without damage.

Sharp‐edge oscillation generates propulsion through localized acoustic streaming around resonantly excited solid microstructures, such as flagella, ciliary bands, or compliant hinges. This mechanism combines structural simplicity, design flexibility, and compatibility with standard microfabrication techniques, enabling multifunctional microswimmers capable of cargo grasping, transport, and release. Frequency‐selective actuation further allows independent control of different structural elements within a single device. However, streaming velocity is fundamentally limited by oscillation amplitude and fluid viscosity. Moreover, achieving true 3D navigation remains a major challenge, as most existing designs are restricted to planar motion. Extending this strategy to fully 3D operation will require either three‐dimensional flagellar architectures or spatiotemporally modulated acoustic fields.

### 4.4 Microrobot Swarm

Microrobots can operate in highly confined environments, but individual units are inherently limited by low propulsive force, restricted manipulation capability, and susceptibility to environmental perturbations. These constraints have motivated increasing interest in cooperative behaviors and swarm‐level functionality [330, 331, 332]. As discussed in Section 4.1, standing wave fields can induce autonomous motion and emergent collective dynamics of active particles [269, 333, 334]. More broadly, engineered acoustic pressure landscapes enable programmable aggregation and dispersion of microswimmers [302, 335], while inter‐particle interactions provide a basis for tunable collective behavior [336]. For example, bimetallic nanorods with Ni segments exhibit self‐propulsion in standing wave fields and spontaneously assemble into dimers and higher‐order structures through magnetic interactions, with aggregate size governed by entropic effects [337]. Magnetically assembled clusters can further function as reconfigurable microrobots, enabling on‐demand assembly within artificial vasculature and upstream locomotion along vessel walls [338, 339]. A representative swarm system achieves reversible, magnetically controlled self‐assembly, allowing dynamic tuning of cluster size relative to the acoustic trapping threshold and eliminating reliance on gravity‐mediated contact forces, with rolling speeds of 10–20 µm s^−^^1^ in static microfluidic environments (Figure 24A) [340]. Subsequent work identified the Mason number (ratio of viscous to magnetic forces) [341, 342] as a key design parameter for optimizing swarm morphology and shear‐flow stability, and established a lubrication‐based dynamical model linking upstream velocity to magnetic rotation frequency, swarm size, and flow conditions (Figure 24B) [343]. Beyond boundary‐assisted locomotion, recent studies demonstrate symmetry‐breaking propulsion in unbounded fluids. Microchain assemblies can achieve rolling motion in open liquid environments, where time‐dependent offsets between rotational and geometric center generate asymmetric acoustic radiation torques that drive propulsion (Figure 24C) [344]. This mechanism eliminates the need for physical boundaries and broadens the operational scope of microrobot swarms.

**FIGURE 24:**
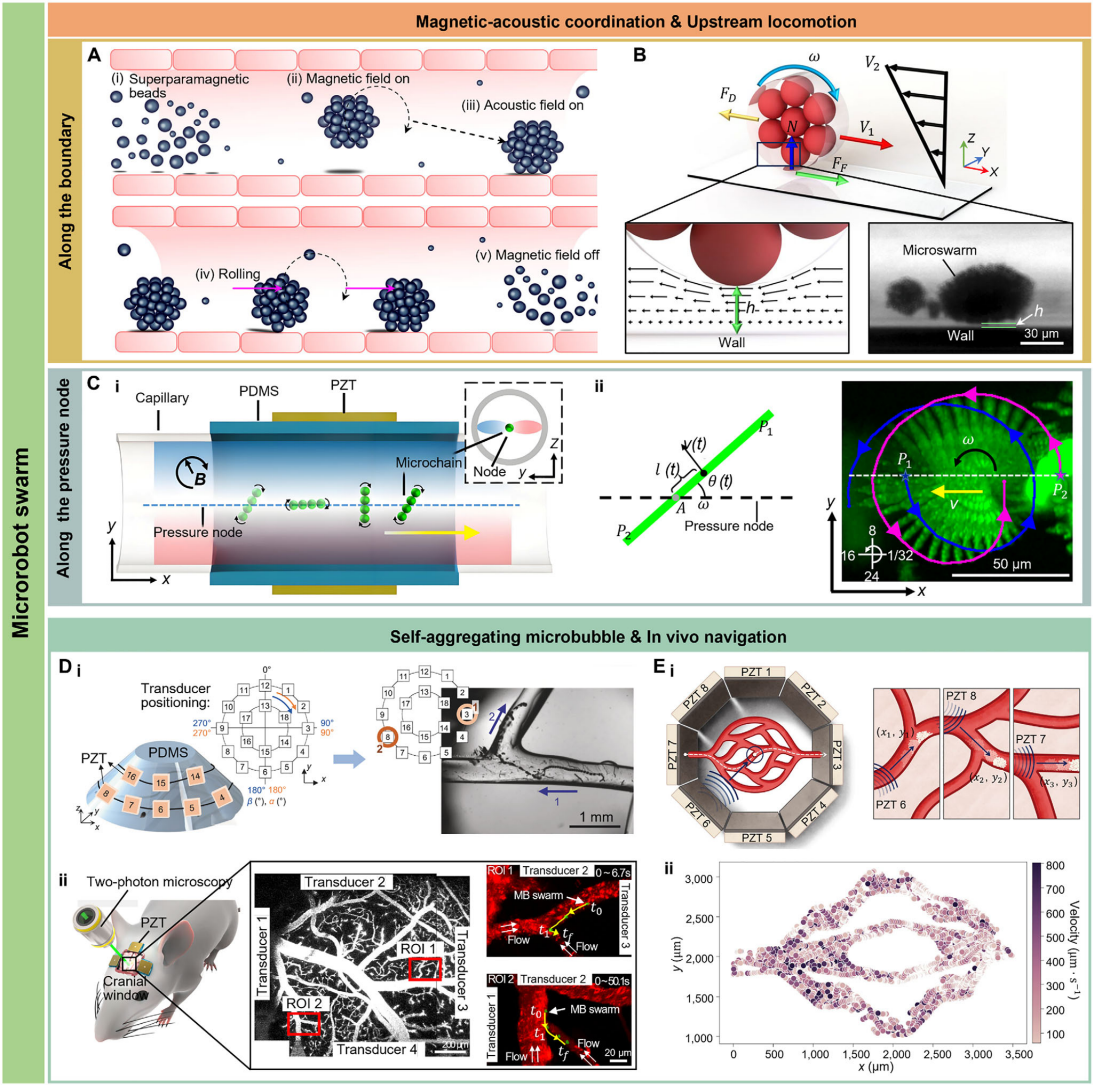
(A) A rolling‐type motion along the boundary: superparamagnetic particles aggregate due to dipole–dipole interaction in the presence of a magnetic field, and the resulting aggregate migrates toward the wall due to the radiation force in an acoustic field. Reproduced with permission [340]. Copyright 2017, Springer Nature. (B) The schematic shows the forces and torques acting on a rolling swarm. The inset illustrates the torque generated by the lubrication layer between the spinning cluster and the wall. Reproduced with permission [343]. Copyright 2021, Springer Nature. (C) (i) Schematic of the experimental setup. The microchain is trapped at the pressure node of the standing wave field. (ii) Schematic of the theoretical model (left). Superimposed time‐lapse images of a microchain undergoing one rotational cycle (right). Under a 1D standing wave field, the rotating microchain exhibits not only off‐center rotation but also a net displacement along the pressure nodal. Reproduced with permission [344]. Copyright 2022, Springer Nature. (D) (i) PDMS cone‐frustum device with two rows of 18 transducers (left). Microscope images show swarm navigation through the fabricated vascular network under combinatorial transducer activation, with an inset schematic indicating the activated transducer for each trajectory (right). (ii) In vivo setup: a cranial window enables two‐photon imaging. Piezoelectric transducers mounted on the skull deliver acoustic waves to steer swarms through branched cerebral vessels. Reproduced with permission [111]. Copyright 2023, Springer Nature. (E) (i) Octagonal arrangement of eight PZTs around an artificial vascular channel (left). The schematic illustrates swarm behavior and ultrasound‐based manipulation principles (right). (ii) Heat map of swarm speed versus position in the artificial channel, with color intensity reflecting local speed. Reproduced with permission [346]. Copyright 2025, Springer Nature.

In parallel, acoustically assembled microbubble clusters have emerged as versatile swarm units capable of navigating complex biological environments [112, 113]. Using orthogonally arranged transducers combined with real‐time two‐photon imaging, programmable assembly, flow‐resistant propulsion, and branch‐selective navigation of microbubble clusters have been demonstrated in cerebrovascular networks (Figure 24D), providing the first in vivo evidence of robust upstream motion and wall adhesion in the brain [111]. However, challenges remain in achieving fully closed‐loop control, deep‐tissue visualization, and scalable multi‐cluster coordination. To address these limitations, model‐based reinforcement learning frameworks (for example, Dreamer v.3) [345] have been introduced, enabling rapid sim‐to‐real transfer and intelligent navigation of ultrasound‐driven microrobots, with success rates approaching 90% (Figure 24E) [346]. These advances mark a transition from task‐specific demonstrations toward adaptive, intelligent swarm systems, with promising implications for targeted drug delivery, precision cell manipulation, and in vivo microrobots.

Overall, microrobot swarms represent a paradigm shift from individual actuation to collective functionality, enabling tasks such as targeted embolization, upstream navigation, and deep‐tissue delivery that are beyond the capabilities of individual microrobots. Table 8 summarizes representative swarm platforms, including their actuation strategies, collective behaviors, and translational progress. Despite these advances, several key challenges remain. First, the mechanisms governing the transition from individual to collective behavior are still poorly understood, particularly the competition and cooperation among Bjerknes, hydrodynamic, and magnetic interactions under acoustic excitation. Second, current real‐time imaging and closed‐loop control capabilities remain inadequate for dynamic in vivo environments. Third, large‐scale implementation is hindered by the lack of standardized fabrication and characterization protocols. Future progress will depend on integrating machine learning‐based adaptive control, advanced real‐time imaging, and multiscale modeling to predict and regulate emergent swarm behaviors.

**TABLE 8:** Summary of representative microswimmer swarms.

Swarm type	Building blocks	Actuation	Key behaviors	Applications	Refs.
Magneto‐acoustic swarms	Magnetic microparticles	Rotating magnetic field and traveling wave field	Rolling‐type motion along boundaries	Upstream navigation in vessels	[340, 343]
Magneto‐acoustic swarms	Magnetic microparticles	Rotating magnetic field and standing wave field	Off‐center rotation, net displacement along pressure nodal line	Rolling motion without physical boundaries	[344]
Acoustic microbubble swarms	Microbubbles (1.1–1.4 µm in diameter)	Transducer arrays (traveling wave field)	Branch‐selective navigation	In vivo navigation in mouse brain vasculature	[111]
Acoustic microbubble swarms	Microbubbles (2–5 µm in diameter)	Transducer arrays (traveling wave field)	Autonomous closed‐loop navigation (model‐based reinforcement learning)	Sim‐to‐real transfer, adaptive navigation	[346]

## 5 Conclusion and Outlook

Acoustic manipulation has emerged as a foundational strategy for particle control and microswimmer propulsion, owing to its non‐contact, label‐free nature, biocompatibility and deep tissue penetration. At the device level, bulk acoustic waves (BAWs) and surface acoustic waves (SAWs) provide the primary platforms for field generation, enabling diverse configurations including focused beams, acoustic vortices and holographic fields. The integration of phased arrays and acoustic metasurfaces has further enabled dynamic and programmable wavefront control, substantially enhancing spatial precision and operational degrees of freedom.

Functionally, acoustic techniques offer broad versatility across gaseous and liquid environments. In air, acoustic levitation enables stable, contactless trapping and reconfigurable positioning of droplets and microparticles. In liquids, acoustic tweezers, streaming and vortex‐based approaches enable multiscale manipulation from nanoparticles to cells, supporting applications in enrichment, separation and patterned assembly (Figures 9, 10, 11, 12, 13, 14, 15, 16, 17, 18). In parallel, acoustic propulsion mechanisms—including shape or density asymmetry, cavitation and sharp‐edge oscillation—enable controlled translation, rotation and collective behaviors of microswimmers, opening new opportunities for targeted drug delivery and minimally invasive intervention (Figures 19, 20, 21, 22, 23, 24). However, translating these proofs‐of‐concept demonstrations into practical diagnostic, manufacturing, and clinical applications requires overcoming persistent physical, biological, and engineering challenges.

At the physical level, spatial resolution is fundamentally limited by the acoustic diffraction limit (∼λ/2). Although higher operating and engineered acoustic fields [347] can partially overcome this constraint, they also increase attenuation and reduce penetration depth. Static holographic lenses provide limited reconfigurability, whereas phased arrays offer dynamic field control at the expense of greater system complexity and cost. Emerging strategies, including programmable acoustic metamaterials [348, 349, 350, 351, 352, 353], deep‐learning‐based inverse design [354], and hybrid acoustic–optical–magnetic field coupling [355, 356, 357], offer promising alternatives but introduce additional integration challenges.

At the biological level, acoustic biocompatibility is strongly parameter‐dependent. Acoustic exposure beyond safe operating thresholds may induce thermal effects [358, 359], streaming‐induced shear stresses [360], and cavitation [361, 362], compromising cell viability, membrane integrity, and gene expression. Sonogenetics [363, 364], which uses ultrasound to activate genetically engineered mechanosensitive ion channels, requires particularly precise pressure control because the therapeutic window between effective neuromodulation and adverse biological responses is exceptionally narrow. Table 9 summarizes the principal safety considerations and corresponding mitigation strategies across representative acoustic modalities. In vivo translation presents additional challenges [6, 365]. Most acoustofluidic platforms have been validated in optically transparent, acoustically homogeneous model systems that poorly represent physiological environments. In the vasculature, blood flow may exceed acoustic trapping forces by orders of magnitude; tissue heterogeneity introduces acoustic impedance mismatches that distort acoustic fields; and the formation of protein coronas on acoustically active particles remains poorly understood. Microswimmers must overcome these constraints while maintaining propulsion efficiency, controllability, and cargo capacity—a combination that remains difficult to achieve in current designs. At the engineering level, key challenges include manufacturing reproducibility, dosage control, swimmer retrieval or biodegradation, and regulatory compliance. The field also lacks standardized fabrication protocols, benchmark performance metrics, and comprehensive biocompatibility databases that systematically correlate acoustic parameters with biological responses across different cell types and exposure conditions.

**TABLE 9:** Key safety considerations and mitigation strategies for acoustic manipulation of biological samples.

Risk category	Primary mechanism	Critical parameters	Typical thresholds/observations	Mitigation strategies
Thermal	Viscous dissipation, transducer losses [366]	Frequency, input power, exposure time, duty cycle	Viability declines beyond ±5°C from 37°C; GHz resonators prone to localized heating [367]	Pulsed operation, low duty cycles, thermally conductive substrates, active cooling
Mechanical (shear)	Acoustic streaming‐induced shear stress [368, 369]	Streaming velocity, channel geometry, cell type [358, 370]	Shear stress exceeding a critical threshold induces membrane disruption; steep velocity gradients damage cells [371]	Optimize channel geometry, reduce input power, use pulsed modes, characterize cell‐type sensitivity
Mechanical (cavitation)	Stable cavitation (microstreaming) and inertial cavitation (collapse‐induced shock, jetting) [372]	Acoustic pressure, frequency, presence of gas nuclei [373, 374, 375, 376, 377]	Inertial cavitation causes irreversible membrane damage and apoptosis; thresholds depend on bubble size and pressure amplitude	Avoid cavitation thresholds unless intended; leverage stable cavitation for sonoporation; characterize bubble dynamics [378]
Biochemical	Mechanotransduction, gene expression modulation, oxidative stress [379, 380, 381, 382]	Exposure duration, intensity, cell type	Sub‐lethal exposure alters gene expression and metabolism; apoptosis can occur without cell lysis; potential reactive oxygen species generation	Minimize exposure time, use lowest effective intensity, validate with cell‐type‐specific assays

Looking forward, several research priorities are critical for clinical translation. In the near term, standardized acoustic exposure protocols and cell‐type‐specific safety databases are needed to enable quantitative comparisons across studies. Engineering efforts should focus on chip‐scale integration, low‐cost manufacturing, and closed‐loop control in physiologically relevant in vitro models. Mid‐term priorities include real‐time 3D imaging for navigation in complex environments, validation in large‐animal models, and the development of biodegradable or retrievable microswimmers supported by regulatory‐grade safety assessments. Long‐term goals include fully implantable or ingestible acoustic microsystems with AI‐driven adaptive control for targeted therapy. Ultimately, establishing clinical‐grade manufacturing and quality‐control frameworks will be essential for translating these technologies beyond proof‐of‐concept. Realizing this vision will require close collaboration across physics, materials science, biology, and engineering. Such interdisciplinary integration will ultimately determine whether acoustic manipulation evolves from a specialized laboratory technique into a transformative clinical and industrial platform.

## Author Contributions

**Jiahui Chu**: investigation; formal analysis; writing – original draft. **Lemin Zhang**: writing – original draft; validation. **Xu Wang**: validation; writing – original draft. **Wenzong Li**: validation; writing – original draft. **Yahua Liw**: formal analysis; investigation; writing – review editing; supervision; project administration.

## Funding

National Natural Science Foundation of China (52475294), the State Key Laboratory of High‐performance Precision Manufacturing (ZY202404), and the Fundamental Research Funds for the Central Universities (DUT24YG133).

## Conflicts of Interest

The authors declare no conflicts of interest.

## Data Availability

The data that support the findings of this study are available from the corresponding author upon reasonable request.
